# Carbohydrates from *Pseudomonas aeruginosa* biofilms interact with immune C-type lectins and interfere with their receptor function

**DOI:** 10.1038/s41522-021-00257-w

**Published:** 2021-12-08

**Authors:** Sonali Singh, Yasir Almuhanna, Mohammad Y. Alshahrani, Douglas W. Lowman, Peter J. Rice, Chris Gell, Zuchao Ma, Bridget Graves, Darryl Jackson, Kelly Lee, Rucha Juarez, Janice Koranteng, Sirina Muntaka, Daniel A. Mitchell, Ana C. da Silva, Farah Hussain, Gokhan Yilmaz, Francesca Mastrotto, Yasuhiko Irie, Paul Williams, David L. Williams, Miguel Cámara, Luisa Martinez-Pomares

**Affiliations:** 1 School of Life Sciences, Faculty of Medicine and Health Sciences, University of Nottingham, Nottingham, NG7 2RD UK; 2 Department of Clinical Laboratory Sciences, College of Applied Medical Sciences, Shaqra University, P.O. Box 33, Shaqra, 11961 Saudi Arabia; 3 Department of Surgery and Center of Excellence in Inflammation, Infectious Disease and Immunity, Quillen College of Medicine, East Tennessee State University, Johnson City, TN 37614 USA; 4 University of Colorado Skaggs School of Pharmacy and Pharmaceutical Sciences, University of Colorado Anschutz Medical Campus, 12850 East Montview Boulevard C238, Aurora, CO 80045 USA; 5 WMS - Translational Medicine, University of Warwick, Coventry, CV4 7AL UK; 6 University of Nottingham Biodiscovery Institute, Nottingham, NG7 2RD UK; 7 School of Pharmacy, University of Nottingham, Nottingham, NG7 2RD UK; 8 National Biofilms Innovation Centre, University of Nottingham, Nottingham, UK; 9 Present Address: Department of Clinical Laboratory Sciences, College of Applied Medical Sciences, King Khalid University, P.O. Box 61413, Abha, 9088 Saudi Arabia; 10 Present Address: University of Central Lancashire, School of Medicine, Vernon Building: VE067, PR1 1JQ Preston, UK; 11 Present Address: Department of Pharmaceutical and Pharmacological Sciences, University of Padova, via F. Marzolo 5, 35131 Padova, Italy; 12 Present Address: Division of Pharmacy and Optometry, University of Manchester, Manchester, UK

**Keywords:** Biofilms, Cellular microbiology

## Abstract

Bacterial biofilms represent a challenge to the healthcare system because of their resilience against antimicrobials and immune attack. Biofilms consist of bacterial aggregates embedded in an extracellular polymeric substance (EPS) composed of polysaccharides, nucleic acids and proteins. We hypothesised that carbohydrates could contribute to immune recognition of *Pseudomonas aeruginosa* biofilms by engaging C-type lectins. Here we show binding of Dendritic Cell-Specific Intercellular adhesion molecule-3-Grabbing Non-integrin (DC-SIGN, CD209), mannose receptor (MR, CD206) and Dectin-2 to *P. aeruginosa* biofilms. We also demonstrate that DC-SIGN, unlike MR and Dectin-2, recognises planktonic *P. aeruginosa* cultures and this interaction depends on the presence of the common polysaccharide antigen. Within biofilms DC-SIGN, Dectin-2 and MR ligands appear as discrete clusters with dispersed DC-SIGN ligands also found among bacterial aggregates. DC-SIGN, MR and Dectin-2 bind to carbohydrates purified from *P. aeruginosa* biofilms, particularly the high molecular weight fraction (HMW; >132,000 Da), with K_D_s in the nM range. These HMW carbohydrates contain 74.9–80.9% mannose, display α-mannan segments, interfere with the endocytic activity of cell-associated DC-SIGN and MR and inhibit Dectin-2-mediated cellular activation. In addition, biofilm carbohydrates reduce the association of the DC-SIGN ligand Lewis^x^, but not fucose, to human monocyte-derived dendritic cells (moDCs), and alter moDC morphology without affecting early cytokine production in response to lipopolysaccharide or *P. aeruginosa* cultures. This work identifies the presence of ligands for three important C-type lectins within *P. aeruginosa* biofilm structures and purified biofilm carbohydrates and highlights the potential for these receptors to impact immunity to *P. aeruginosa* infection.

## Introduction

*Pseudomonas aeruginosa* is a versatile opportunistic pathogen that causes acute infection after invasive procedures and burns, and chronic infections in patients with persistent lung disease and compromised immunity^1^. *P. aeruginosa* infection is especially troublesome in people with cystic fibrosis where it is a major determinant of irreversible loss of lung function and mortality^2,3^. The use of indwelling catheters and implants during hospital procedures, as well as use of contact lenses create niches that are effectively colonised by *P. aeruginosa* which exploits an armoury of cell-associated and secreted virulence determinants that facilitate invasion and establishment of infection^4^.

The transition from planktonic to sessile growth leading to biofilm development are central to *P. aeruginosa* pathogenesis^1,4,5^. Biofilms contribute to *P. aeruginosa* persistence by increasing tolerance to antimicrobial agents and immune defences^1^. Within these bacterial communities, cells are embedded in an extracellular polymeric substance (EPS) matrix mainly composed of polysaccharides, nucleic acids and proteins^6,7^. *P. aeruginosa* produces three major carbohydrates: Psl, Pel and alginate, with Psl and Pel playing major roles in biofilm formation in a strain-dependent manner^6–8^. Psl is neutral and mannose-rich^9^. Pel is cationic and largely composed of *N*-acetyl-galactosamine and *N*-acetyl-glucosamine^10^.

Here, we tested the hypothesis that *P. aeruginosa* biofilms could directly engage lectin receptors expressed by immune cells. Innate immune cells^11^ such as monocytes, macrophages and neutrophils bear C-type lectin receptors (CLRs) and have been implicated in immune responses to *P. aeruginosa* infection^12^. Dendritic cells, which are antigen-presenting cells and instrumental in initiating and modulating adaptive immunity to *P. aeruginosa* infections^13^, also express a host of lectin receptors^11^.

The mannose receptor (MR, CD206)^14^, Dendritic Cell-Specific Intercellular adhesion molecule-3-Grabbing Non-integrin (DC-SIGN, CD209) and dendritic cell-associated C-type lectin-2 (Dectin-2, C-type Lectin domain Family 6, Member A) are CLRs predominantly expressed by populations of myeloid cells^14–16^. MR contains two independent carbohydrate-binding domains, the cysteine-rich domain (MR-CR) and C-type lectin-like domains (MR-CTLDs) that recognise sulfated and mannosylated sugars, respectively^14^. DC-SIGN binds to high mannose structures and blood type Lewis antigens through extracellular CTLD regions clustered through the formation of tetramers^15,17,18^. Neither MR nor DC-SIGN contain bespoke intracellular signalling motifs and rather than directly inducing cellular activation, modulate signalling triggered by other receptors such as Toll-like receptors^19^ and Fc receptors^14,20^. Dectin-2 is a transmembrane type II protein containing a single extracellular CTLD^16,21^. Dectin-2 lacks signalling motifs but induces an activating signal by engaging the adaptor molecule Fc receptor common γ chain^16,22^. Dectin-2 specifically binds glycoconjugates bearing the disaccharide Manα1–2-Man including fungal α-linked mannans, *Malassezia* glycoproteins, and bacterial lipopolysaccharides^23^. The contribution of MR, DC-SIGN and Dectin-2 to immunity against infection is complex. MR and DC-SIGN bind a wide range of pathogens. DC-SIGN engagement has been associated with immunomodulation^24,25^ while MR seems to play a redundant role during infection^14^ and has been implicated in immunosuppression^26^ as well as triggering cellular signalling through FcR common γ chain^27^. Dectin-2 mediates immune activation in response to infection with fungi, *Schistosoma mansoni* and bacteria^28–32^.

Here, we demonstrate that DC-SIGN, MR and Dectin-2 recognise *P. aeruginosa* biofilms and purified biofilm carbohydrates. In addition, DC-SIGN binds planktonic *P. aeruginosa* cultures and this binding depends on the presence of the common polysaccharide antigen (CPA) of lipopolysaccharide (LPS)^33^. We also show that purified biofilm carbohydrates interfere with the activity of all three receptors and influence the biology of monocyte-derived dendritic cells (moDCs). These results suggest a potential role for C-type lectins in modulating immune responses during *P. aeruginosa* biofilm-driven chronic infections.

## Results

### Analysis of DC-SIGN, MR, and Dectin-2 binding to *P. aeruginosa* biofilms

During infection, biofilm formation can alter the range and distribution of bacterial molecular patterns presented to innate immune receptors^34^. Carbohydrates are major components of *P. aeruginosa* biofilms^8^ and strategically posed to engage lectin receptors, a subset of pattern recognition receptors specialised in sugar recognition. In particular, the high mannose content of Psl suggested potential binding to mannose-binding CLRs such as DC-SIGN, MR and Dectin-2. We queried whether *P. aeruginosa* biofilms are directly recognised by DC-SIGN, MR and Dectin-2 by analysing the interaction of recombinant Fc-chimeric molecules DC-SIGN-Fc, MR-CTLD4-7-Fc^35^ and Dectin-2-Fc with biofilms generated by PAO1^36^ and the exopolysaccharide mutants: Δ*wspF* (Psl+ /Pel+)^37^, Δ*wspF* Δ*pel* (Psl+ /Pel−) and Δ*wspF* Δ*psl* (Psl−/Pel+)^38^ and wells containing cultures of the Δ*wspF* Δ*psl* Δ*pel* (Psl−/Pel−) mutant^39^ which is biofilm-deficient (Table 1). The Δ*wspF* background confers constitutive high levels of cyclic-di-GMP which in turn increases the expression of the Psl and Pel biosynthetic genes and biofilm formation^40^. Furthermore, Psl stimulates a further increase in cyclic-di-GMP levels acting as a feedback loop in the stimulation of biofilm formation^7^.

**Table 1:** Strains used in this study.

Strain	Features	Reference
PAO1	WT Physiological levels of c-di GMP Produces Psl and Pel	^36^
*∆wspF*	High cellular levels of c-di GMP Produces Psl and Pel	^37^
∆*wspF*∆*pel*	High cellular levels of c-di GMP Produces high levels of Psl Lacks Pel	^38^
∆*wspF*∆*psl*	High cellular levels of c-di GMP Produces high levels of Pel Lacks Psl	
∆*wspF*∆*pel*∆*psl*	High cellular levels of c-di GMP Lacks Psl and Pel Biofilm deficient	^39^
Δ*wbpM*	CPA^+^/OSA^−^	^41^
Δ*rmd*	CPA^−^/OSA^+^	
Δ*wbpL*	CPA^−^/OSA^−^	

**Fig. 1:**
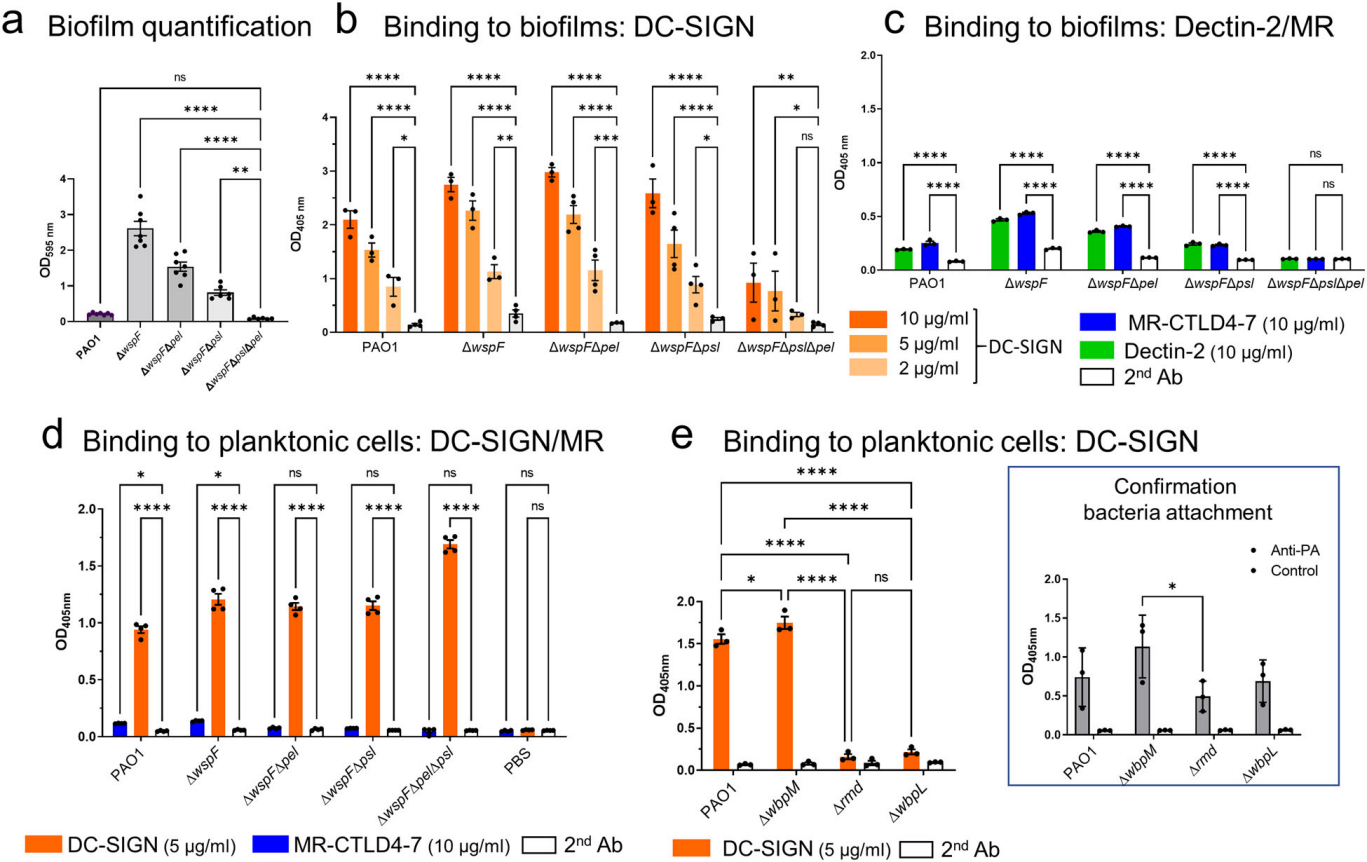
Binding of C-type DC-SIGN, MR and Dectin-2 to *P. aeruginosa* biofilms and planktonic cells. **a** PAO1, Δ*wspF* (Psl + /Pel+), Δ*wspF* Δ*pel* (Psl + /Pel-), Δ*wspF* Δ*psl* (Psl-/Pel+) and Δ*wspF* Δ*psl* Δ*pel* (Psl-/Pel-) were grown in 96-well plates for 24 h in X-Vivo-15 and biofilm formation was analysed using the crystal violet assay. Data analysed using One-way ANOVA and corrected for multiple comparisons using Dunnett’s multiple comparison test. *N* = 6-7 in triplicate. ** 0.001; **** <0.0001. **b** and **c**. PAO1, Δ*wspF*, Δ*wspF* Δ*pel* or Δ*wspF* Δ*psl* biofilms were generated in 96-well plates for 24 h, fixed and incubated with DC-SIGN-Fc (B) or MR-CTLD4-7-Fc or Dectin-2-Fc (C) followed by anti-human Fc secondary antibody conjugated to alkaline phosphatase. Cultures of Δ*wspF* Δ*psl* Δ*pel* were used as controls. Analysis performed using Two-way ANOVA corrected for multiple comparisons using the Dunnett’s multiple comparisons test. *N* = 3 in triplicate. In B * 0.0324 (for Δ*wspF* Δ*psl*); 0.0413 for Δ*wspF* Δ*psl*Δ*pel;* ** 0.007*;* *** 0.0007*;* **** <0.0001. In C **** <0.0001. **d** Planktonic cultures of *P. aeruginosa* PAO1 and the different mutants were collected, fixed and used to coat wells of MaxiSorp plates. Wells were incubated with MR-CTLD4-7-Fc or DC-SIGN-Fc followed by anti-human Fc secondary antibody conjugated to alkaline phosphatase. DC-SIGN, but not MR, bound planktonic bacteria and binding was independent of the presence of Psl and/or Pel. Two-way ANOVA corrected for multiple comparisons using the Dunnett’s multiple comparisons test. *N* = 4 in triplicate. *, 0.0412 for PAO1 and 0.0148 for Δ*wspF*; ****, <0.0001.**e**. DC-SIGN binding to planktonic bacteria was dependent on the presence of CPA LPS which is absent in the Δ*rmd* and Δ*wbpL* mutants. *N* = 3 in triplicate. *, 0.0111; ****, <0.0001. Right panel: Adherence of planktonic cells to the wells was confirmed by ELISA using an antibody against *P. aeruginosa* (Anti-PA). *N* = 3 in triplicate. *, 0.0161. Analysis performed using Two-way ANOVA corrected for multiple comparisons using the Tukey’s multiple comparisons test. Graphs show mean +/− SEM. Specificity controls for these assays are shown in Fig. S1. In all cases, reading were taken within 20 min development and all strains in each graph were tested simultaneously.

For our study, all biofilms were generated in serum-free eukaryotic media (X-Vivo-15) to mimic in vivo conditions for 24 h. In this setting, we observed robust biofilm formation by all Δ*wspF* mutants expressing Psl and/or Pel while the PAO1 wild-type biofilms consistently displayed low biomass (Fig. 1a). We identified strong binding of DC-SIGN to all biofilm cultures, including PAO1 and the Δ*wspF* Δ*psl* (Psl−/Pel+) mutant, but variable, weak binding to wells with Δ*wspF* Δ*psl* Δ*pel* (Psl−/Pel−) mutant cultures (Fig. 1b). MR-CTLD4-7 and Dectin-2 bound to all biofilm cultures including PAO1 and the Δ*wspF* Δ*psl* (Psl–/Pel+) mutant but the binding was limited in all instances compared to DC-SIGN (Fig. 1c). Further analysis demonstrated preferential inhibition of DC-SIGN binding to PAO1 biofilms by mannose and fucose compared to galactose (Supplementary Fig. 1).

Due to the high binding of DC-SIGN to Δ*wsp*F Δ*psl* biofilms and weak binding to Δ*wspF* Δ*psl* Δ*pel* cultures, we investigated whether DC-SIGN could also interact with planktonic cells. DC-SIGN recognised *P. aeruginosa* planktonic cells and binding was independent of Psl and/or Pel (Fig. 1d). Here bacteria attachment to wells was independent from biofilm formation. *P. aeruginosa* produces two forms of LPS O-antigen; a homopolymer of D-rhamnose trisaccharide repeats named CPA or A band, and a heteropolymer that consists of repeating units of three to five distinct sugars named O-specific antigen (OSA) or B band^33^. Deletions in *wbpM*, *rmd*, or *wbpL* in PAO1 causes loss of OSA, CPA, or both, respectively^41^. Binding of DC-SIGN to planktonic *P. aeruginosa* required expression of *rmd* or *wbpL* (Fig. 1e) suggesting the requirement for CPA LPS. Preliminary imaging results confirmed DC-SIGN binding to planktonic PAO1 (Supplementary Fig. 2). No Dectin-2 or MR binding to planktonic cells was observed (Fig. 1d and Supplementary Fig. 2).

To determine whether C-type lectin recognition of *P. aeruginosa* biofilms could be extended to the clinical setting we analysed binding of DC-SIGN, MR and Dectin-2 to biofilms generated by several wound isolates (Supplementary Figs. 3 and 4). Results showed a binding pattern similar to that found for the laboratory strains, with consistently high levels of binding by DC-SIGN compared to MR and Dectin-2 which bound weakly.

These results demonstrate that *P. aeruginosa* biofilms produced by laboratory and clinical isolates display ligands for C-type lectins with ligands for DC-SIGN being more abundant and/or accessible. Ligands for DC-SIGN are also present in planktonic cells and depend on the presence of CPA LPS but not on Psl and/or Pel expression.

### Spatial distribution of DC-SIGN, MR, and Dectin-2 ligands within PAO1 biofilms

**Fig. 2:**
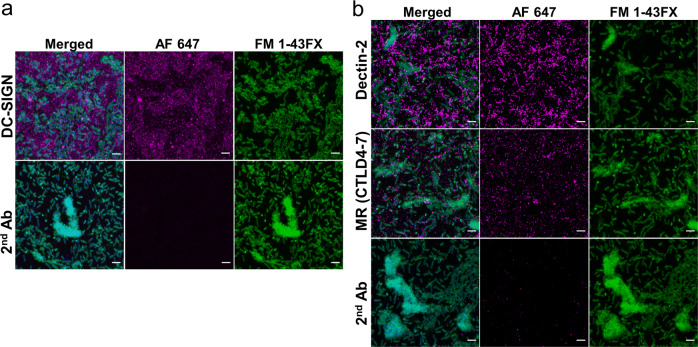
Distribution of DC-SIGN, MR and Dectin-2 ligands in *P. aeruginosa* biofilms. PAO1 biofilms generated underflow conditions for 18 h as described in materials and methods were incubated with DC-SIGN, MR-CTLD4-7 and Dectin-2 Fc-chimeric proteins followed by anti-human Fc secondary antibody conjugated to Alexa 647 (magenta) and counterstained with DAPI (DNA, blue) and FM1-43FX (bacteria, green). Z-stacks were acquired for all samples using confocal microscopy. The figure shows 3D projections for the three receptors based on the initial detection of bacterial cells during image acquisition. The same settings for image acquisition and processing were maintained for test and control samples. Scale bar is 4 µm. Brightness and contrast settings in Fiji for DC-SIGN (**a**) and corresponding secondary antibody control (2nd Ab) (AF647 channel): minimum and maximum values 48 and 1497, respectively. For MR and Dectin-2 (**b**) and corresponding 2nd Ab control (AF647 channel): 330 for both. Settings for FM 1-43 FX and DAPI channels were the same in all instances. All slices for the three receptors are shown in Supplementary Fig. S5.

To determine the location of DC-SIGN, MR and Dectin-2 ligands within *P. aeruginosa* biofilms, we analysed the binding of DC-SIGN, MR and Dectin-2 Fc proteins to biofilms generated under flow conditions in X-Vivo-15 medium using confocal microscopy. This approach unveiled unique ligand distribution for each lectin (Fig. 2). DC-SIGN ligands were very abundant and dispersed among bacteria aggregates, reaching substantial density in some areas located in the upper regions of the biofilm (Fig. 2a and Supplementary Fig. 5, top panels). MR-CTLD4-7 and Dectin-2 ligands were less abundant (laser intensity was increased for their detection) and displayed a more granular distribution (Fig. 2b and Supplementary Fig. 5). Binding was particularly bright for Dectin-2 compared to MR which contrast with the ELISA results when both lectins display similar binding. These differences could be caused by the use on an alternative detection system or changes in carbohydrate structure when biofilms are formed under flow conditions. Together these results support the display of DC-SIGN, MR and Dectin-2 ligands by *P. aeruginosa* biofilms, with each receptor exhibiting different binding profiles.

### DC-SIGN, MR, and Dectin-2 bind to carbohydrates produced by *P. aeruginosa* biofilms

After detecting ligands for DC-SIGN, MR and Dectin-2 in *P. aeruginosa* biofilms, it was important to establish whether these receptors recognised polysaccharides produced during biofilm formation. Towards this aim, we purified carbohydrates from biofilm cultures of Δ*wspF* Δ*pel* (Psl + /Pel-) (Table 1) to enrich for mannose-rich polysaccharides as previously described^42^. Two independent preparations, 1 and 2, were generated and divided into high (>45 kDa, HMW) and low molecular weight (<45 kDa, LMW) fractions by gel filtration chromatography based on protein standards^42^. We employed several approaches to investigate the binding of these carbohydrate preparations to DC-SIGN, MR and Dectin-2. In the first instance, we tested the binding of Fc constructs to wells coated with different concentrations of HMW and LMW (Supplementary Fig. 6). In all instances, dilution of LMW preparations from 10 to 0.5 µg/ml had a higher impact on lectin binding than dilution of the HMW preparations which was indicative of the presence of more ligands for these CLRs in the HMW fractions (Supplementary Fig. 6a). Further dilution of HMW-1 and HMW-2 fractions confirmed dose-dependent binding for DC-SIGN and MR at 0.5 to 0.02 µg/ml (Supplementary Fig. 6b).

Gel permeation chromatography (GPC) confirmed differences in molecular weight between the HMW and LMW preparations (15,370 Da for LMW-1 and 182,300 Da and 132,670 Da, for HMW-1 and HMW-2, respectively. LMW-2 size was not investigated) (Supplementary Fig. 6c). A substantial amount of the material in all the samples (~33–40% of the total mass) eluted with the included volume. In our system, this means compounds with low MW, i.e. 1000 Da. Their nature is unknown, but we propose that they are carbohydrate breakdown products S6C.

Initial ^1^H NMR analysis indicated increased level of impurities in LMW-1 compared to HMW-1 and HMW-2 (data not shown), hence further work focused on HMW preparations. The hydrolysed carbohydrate monomer compositions in weight % for HMW-1 is 74.9% mannose, 14.7% glucose, 7.4% galactose, and 3.0% rhamnose, and for HMW-2 80.9% mannose, 11.0% glucose, 2.3% galactose, and 5.7% rhamnose. The ^1^H NMR spectra of HMW-1 and HMW-2 are very similar (Supplementary Fig. 7a) and indicate that mannose, the major monomer present, arose from mannan segments in the polymer^43^.

**Fig. 3:**
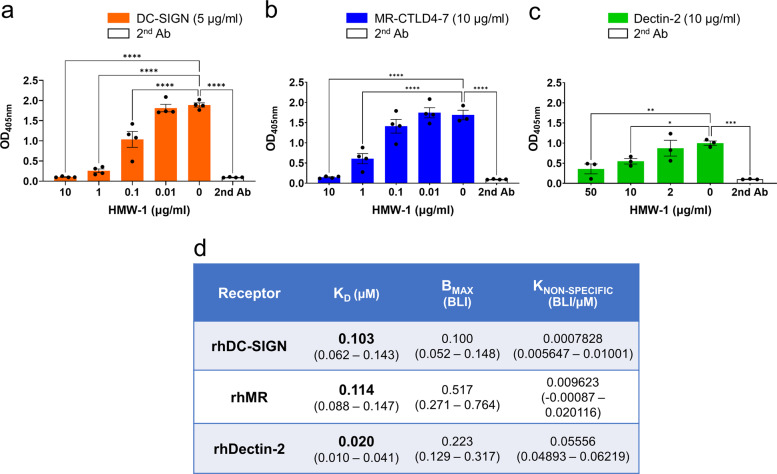
Binding of DC-SIGN, MR-CTLD4-7 and Dectin-2 to *P. aeruginosa* biofilm carbohydrates. **a**–**c**. Lectin competition assays demonstrate dose-dependent inhibition of fucose-PAA (2 µg/ml) binding to DC-SIGN (**a**) and MR-CTLD4-7 (**b**), and of zymosan (1 × 10^6^ particles) to Dectin-2 (**c**), by HMW-1. Fc-chimeric proteins and anti-human Fc-secondary antibody conjugated to alkaline phosphatase were used. Graphs show mean ± SEM of 4 independent repeats done in duplicate. Analysis was performed using One-way ANOVA corrected for multiple comparisons using the Dunnett’s multiple comparisons test. ****, <0.0001; ***, 0.0007; **, 0.007, *, 0.0493. **d** HMW-2 bin**d**s rhDC-SIGN, rhMR, and rhDectin-2. Tetrameric hDC-SIGN, biotinylated and immobilised on a streptavidin sensor and rhMR and rhDectin-2 immobilised on a Ni-sensor were incubated with different HMW-2 concentrations. The table shows equilibrium dissociation constants for the receptor ligand interaction in μM (K_D_); receptor density on the biosensor surface (B_MAX_) and nonspecific binding (K_NON-SPECIFIC_). 95% confidence intervals in µM are shown within brackets.

To complement previous assays, we tested the ability of HMW-1 to compete for binding of DC-SIGN, MR and Dectin-2 to known ligands. For this Fc proteins were preincubated with different concentrations of HMW-1 and then added to wells coated with fucose-PAA (DC-SIGN and MR) or zymosan (Dectin-2) (Fig. 3). We observed dose-dependent inhibition in all instances with HMW-1 significantly inhibiting fucose-PAA binding by DC-SIGN from 0.1 µg/ml (Fig. 3a) and by MR from 1 µg/ml (Fig. 3b). HMW-1 inhibited the binding of Dectin-2 to zymosan from 10 µg/ml (Fig. 3c).

The binding of DC-SIGN, MR and Dectin-2 to biofilm carbohydrates was further confirmed using biotinylated tetrameric DC-SIGN^18^, full-length human MR and Dectin-2, and HMW-2 and biolayer interferometry (BLI). Analysis of the binding kinetics revealed that all three receptors bound HMW-2 with K_D_s in the nM range (Fig. 3d). In this experimental setting, the K_D_ for Dectin-2 binding was lower (20 nM K_D_) compared to DC-SIGN and MR (103 nM and 114 nM K_D_, respectively). See Supplementary Fig. 8 for a representative sensorgram of the interaction of Dectin-2 with HMW-2.

In conclusion, several experimental approaches support the interaction of DC-SIGN, MR and Dectin-2 with polysaccharides from *P. aeruginosa* biofilms. All three receptors bound biofilm carbohydrates attached to plastic (Lectin ELISAs) and in solution (competition ELISAs and BLI). Further, binding of MR and Dectin-2 was observed with both human and murine versions of the receptors (murine receptor-Fc constructs used in the ELISA binding assays and His-Tagged human receptors used in BLI assays). For DC-SIGN, Fc and biotinylated tetrameric protein showed binding. We propose that discrepancies among assays regarding the strength of these interactions are likely caused by differences in ligand and receptor clustering among assays, which will affect binding avidity^44^.

### Biofilm carbohydrates interfere with the endocytic activity of cell-associated DC-SIGN and MR

**Fig. 4:**
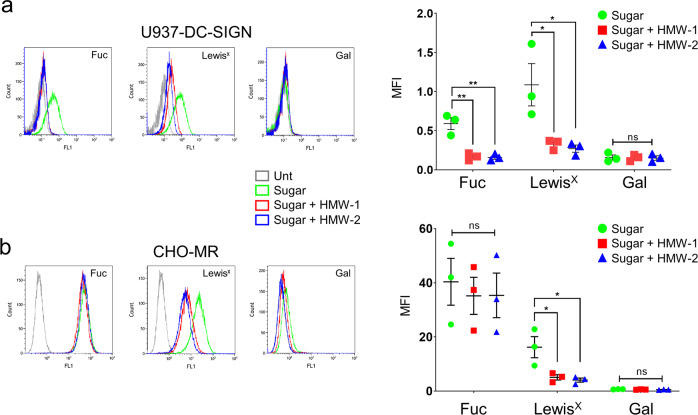
Inhibition of polymeric ligand uptake by DC-SIGN and MR expressing cells by HMW-1 and HMW-2. Cells were treated with HMW-1 or HMW-2 (10 µg/ml) for 1 h, then fluorescein-labelled polymeric ligands were added for a further 1 h. The association of fluorescent polymeric ligands with cells was measured by flow cytometry. Representative histograms and scatter plots depicting mean ± SEM of median fluorescence intensity (MFI) are shown for each cell type. **a** U937-DC-SIGN cells (express DC-SIGN, but not MR) associated with fucose and Lewis^x^ polymers and this association was reduced by HMW-1 and HMW-2, *N* = 3. **b** CHO-MR cells (express MR, but not DC-SIGN) associated with fucose and Lewis^x^ polymers and only Lewis^x^ association was reduced by HMW-1 and HMW-2, *N* = 3. One-way ANOVA corrected for multiple comparisons using the Tukey’s multiple comparisons test. *, ≤0.05. ** ≤0.01. Fuc fucose, Gal galactose.

To further analyse the interaction between biofilm carbohydrates and DC-SIGN, MR and Dectin-2, we tested whether these polysaccharides could impact on receptor activity. First, we investigated the ability of HMW-1 and HMW-2 to interfere with the activity of cell-associated DC-SIGN and MR using the stable transfectants U937-DC-SIGN and CHO-MR^45^, respectively. We confirmed the expression of DC-SIGN in U937-DC-SIGN using flow cytometry (Supplementary Fig. 9a). U937-DC-SIGN cells associate with the model polymeric ligands Lewis^x^-PAA-FITC and fucose-PAA-FITC. Initial results showed inhibition of Lewis^x^ association by HMW-1 in a dose-dependent manner (50-0.4 µg/ml, Supplementary Fig. S9B) and that 10 µg/ml provided good inhibition. Based on these findings we investigated fucose and Lewis^x^ association to U937-DC-SIGN in the presence and absence of HMW-1 and HMW-2 (10 µg/ml). Results showed reduced association of both ligands in the presence of biofilm polysaccharides (Fig. 4a), indicating that these carbohydrates can compete with both DC-SIGN ligands for binding.

Fucose-PAA-FITC and, weakly, Lewis^x^-PAA-FITC associated with CHO-MR cells but not with control CHO cells (Supplementary Fig. S9c). The association of Lewis^x^-PAA-FITC to MR-expressing cells was unexpected as the MR-CTLD4-7 fragment does not bind Lewis^x^ in ELISA-based assays (Supplementary Fig. 1) and this polymeric ligand lacks the sulfated moiety required for binding to MR-CR domain^46^. Surface plasmon resonance (SPR) analysis using full-length human MR suggests weak unspecific binding of MR to Lewis^x^ polymers (Supplementary Fig. 10). HMW-1 and HMW-2 reduced the association of Lewis^x^-PAA-FITC, but not fucose-PAA-FITC, to CHO-MR cells. We previously observed good inhibition of ligand association by a known positive control for MR (mannan) in these cells indicating that inhibition by a MR ligand is feasible in this model^47^. These results suggest that biofilm carbohydrates interfere with the binding of selected MR ligands, but possibly only those with very low binding avidity.

These results were reproduced with an HMW-2 preparation lacking LPS contamination (Supplementary Fig. 11) indicating that LPS does not contribute to this inhibitory activity. ELISA-based assays confirmed binding of LPS-free HMW-2 to DC-SIGN and MR (Supplementary Fig. 12). Together these findings indicate that biofilm carbohydrates compete with known ligands for cell-associated DC-SIGN, leading to reduced cellular binding. Findings for MR indicate weaker competition.

### Biofilm carbohydrates act as Dectin-2 antagonists

The high-affinity interaction between the Dectin-2 and HMW-2 suggested the possibility of biofilm polysaccharides triggering cellular activation through Dectin-2. Hence, we investigated the ability of Dectin-2 reporter cells (rmDectin-2 HEK cells) to respond to these carbohydrates. LPS-free HMW-2 was used for these studies to minimise potential confounding factors caused by LPS O-antigens. We demonstrated the binding of LPS-free HMW-2 to Dectin-2 which could be inhibited by the Dectin-2 ligand Mannα1-2 Mann (Supplementary Fig. 13). In addition, we examined the binding of LPS-free HMW-2 to rhDectin-2 using BLI. The data support a K_D_ for the interaction of 0.0186 µM (95% CI = 0.0080–0.0433) which is similar to that previously estimated for HMW-2 (Fig. 3d).

**Fig. 5:**
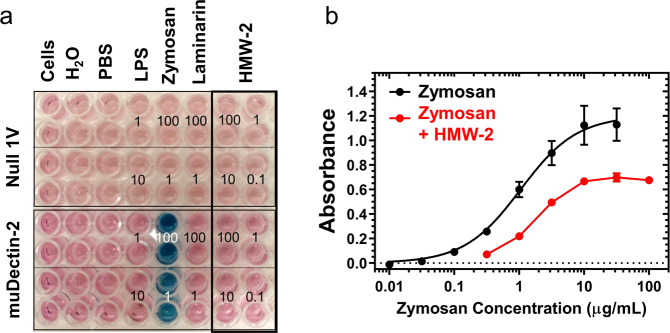
HMW-2 inhibits Dectin-2-mediated cellular activation. **a** HMW-2 does not induce Dectin-2 activation. Dectin-2 reporter cells (HEK-blue muDectin-2) and control cells (Null 1 V) (5 × 10^4^ cells per well) were exposed to different stimuli [LPS (1 and 10 µg/ml), Zymosan (1 and 100 µg/ml), laminarin (100 and 1 µg/ml) and LPS-free HMW-2 (100, 10, 1, and 0.1 µg/ml)] for 18 h. Plates were read at OD_645 nm_. Figure shows representative image from two independent repeats. **b**. HMW-2 acts as an antagonist for Dectin-2. Dectin-2 reporter cells (5 × 10^4^ cells per well) were preincubated with 0.3 µg/ml of LPS-free HMW-2 for 1 h and then exposed to different zymosan doses for 18 h. Plates were read at OD_645 nm_. The figure shows representative results from 3 independent repeats.

Dectin-2 reporter cells were exposed to different concentrations of LPS-free HMW-2 (100, 10, 1, and 0.1 µg/ml) or yeast-derived zymosan particles, as positive controls. Surprisingly, LPS-free HMW-2 failed to activate Dectin-2 reporter cells (Fig. 5a) despite the cells showing a clear response to zymosan particles. An alternative outcome for the interaction between Dectin-2 and biofilm polysaccharides is for these carbohydrates to act as receptor antagonists. To test this possibility Dectin-2 reporter cells were exposed to different concentrations of LPS-free HMW-2 before being stimulated with zymosan. Under these conditions, LPS-free HMW-2 (0.3 µg/ml) inhibited the response of Dectin-2 reporter cells to zymosan (Fig. 5b). Analysis of the inhibition of zymosan response in Dectin-2 reporter cells provides an estimate for an apparent zymosan dissociation constant of 1.89 µg/mL (95% CI 1.57–2.22). Thus, these data suggest that LPS-free HMW-2 is bound by Dectin-2 and does not dissociate from the Dectin-2 receptor, thus preventing zymosan binding and activation. These data are consistent with the binding data for HMW-2 in which there is very little indication of binding dissociation, supporting an interpretation that HMW-2 acts as a non-displaceable antagonist for rmDectin-2 attenuating its stimulation by zymosan.

### Effect of biofilm carbohydrates on monocyte-derived dendritic cells

**Fig. 6:**
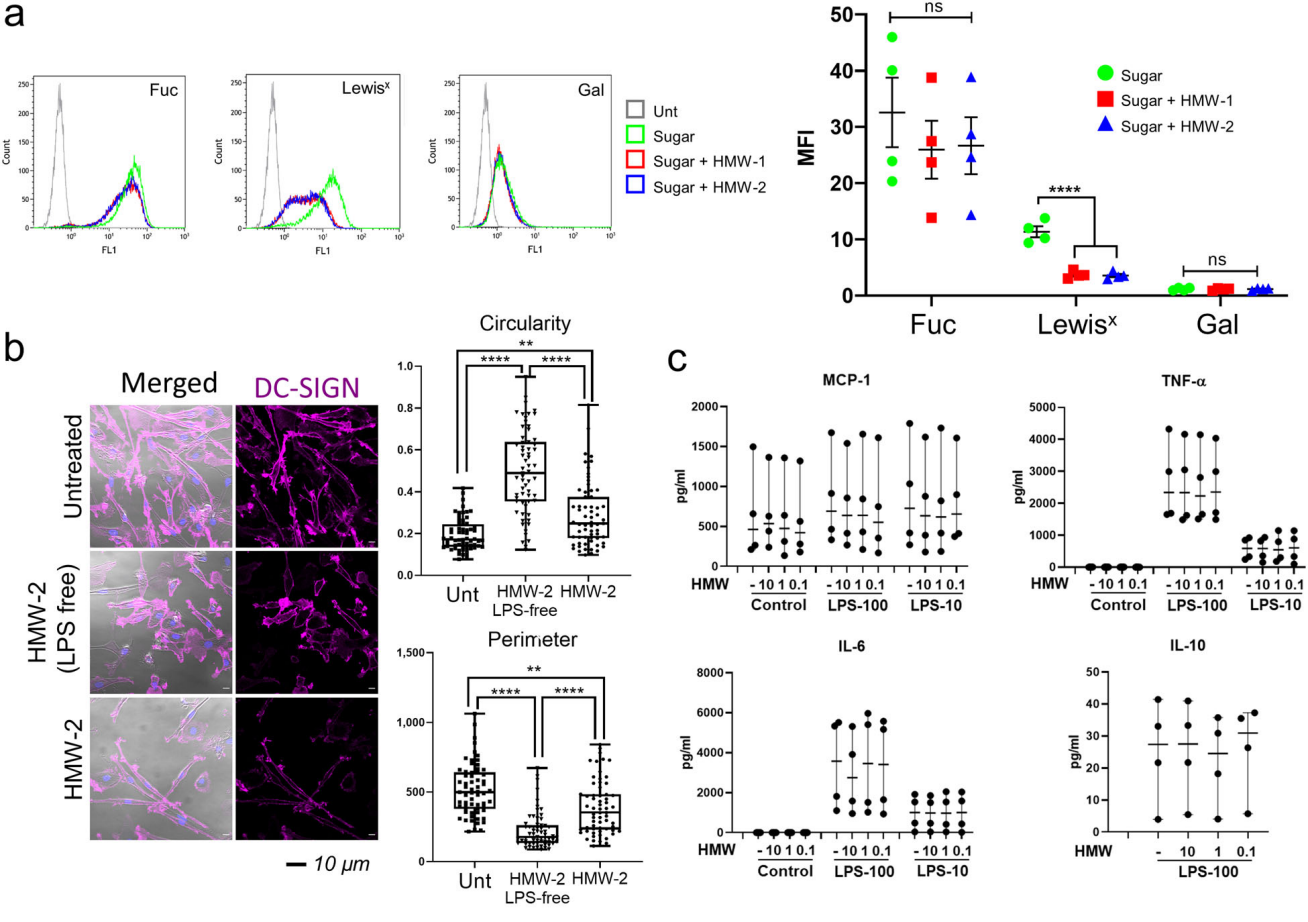
Effect of biofilm carbohydrates on human moDCs. **a** Human moDCs (MR+ and DC-SIGN+) bind fucose (Fuc), Lewis^x^ and galactose (Gal) PAA-FITC polymers but only Lewis^x^ association is reduced by HMW-1 and HMW-2. Cells were treated with HMW-1 or HMW-2 (10 µg/ml) for 1 h, then fluorescein-labelled polymeric ligands were added for a further 1 h. Association of fluorescent polymeric ligands to moDCs was measured by flow cytometry. Representative histograms are shown as well as a graph depicting mean ± SEM of median fluorescence intensity (MFI), *N* = 4. One-way ANOVA corrected for multiple comparisons using the Tukey’s multiple comparisons test. ****, ≤0.0001. Fuc fucose; Gal galactose. **b**. Changes in human moDC morphology in the presence of biofilm-associated carbohydrate. HMW-2 (with and without endogenous LPS, 10 µg/ml, diluted in X-Vivo-15 medium) was used to coat chambers of µ-slide VI 0.4 flow slides overnight at 4 °C. MoDCs were added (5 × 10^4^ cells per channel) and incubated for 24 h. Samples were then fixed and stained for DC-SIGN (magenta) and nucleus (DAPI, blue). The figure shows representative images from unpermeabilised samples. Permeabilised samples, including secondary antibody control are shown in Supplementary Fig. S16. Cells were analysed for changes in shape (Circularity Index), size (Perimeter). Analysis of DC-SIGN labelling intensity (Raw Integrated Density and Signal per Unit Area) is shown in Supplementary Fig. S16. Data derive from 3 independent experiments, 20 cells per experiment were analysed. Statistical significance assessed using Kruskal-Wallis test corrected for multiple comparison using a Dunn’s multiple comparison test. **c**. LPS-free HMW-2 does not affect cytokine production by moDCs in response LPS. MoDCs (10^5^ cells per well) were added to 48 well tissue culture plates coated with different doses of LPS-free HMW-2 (10, 1, and 0.1 µg/ml) for 16 h. Cultures were incubated for 2 h and then treated for 4 h with ultrapure *E. coli* LPS (100 and 10 ng/ml, LPS-100 and LPS-10) and supernatants collected for cytokine quantification. *N* = 4. Controls: samples treated with buffer or incubated only with LPS-free HMW-2. Only samples treated with LPS-100 ng/ml in the presence and absence of LPS-free HMW-2 were analysed for IL-10.

Inhibition of DC-SIGN, Dectin-2 and, partially, MR activities by biofilm carbohydrates can potentially influence the biology of immune cells expressing these receptors. Monocyte-derived dendritic cells (moDCs) offer a good model to study the function of DC-SIGN and MR in human antigen-presenting cells and we consistently detected both receptors under our experimental conditions while Dectin-2 levels were very low in all instances (Supplementary Fig. 14). To determine if biofilm carbohydrates could affect the endocytic activity of moDCs, we investigated the association of selected fluorescein-labelled polymeric ligands with these cells and how this was affected by HMW-1 and HMW-2. MoDCs effectively bind fucose-PAA-FITC and Lewis^x^-PAA-FITC compared to galactose-PAA-FITC (Fig. 6a). When association assays were performed in the presence of HMW-1 and HMW-2, binding of Lewis^x^-PAA-FITC was significantly reduced while that of fucose-PAA-FITC or galactose-PAA-FITC remained unaffected (Fig. 6a). These findings were not influenced by the presence of LPS as Polymyxin B (100 µg/ml) did not alter polymer association in the presence or absence of biofilm polysaccharides (Supplementary Fig. 15). Combined with the previous results using the stable cell lines U937-DC-SIGN and CHO-MR (Fig. 4), these data support the ability of biofilm carbohydrates to interfere with sugar association with moDCs. Our findings also unveil the potential for differential contribution of MR and DC-SIGN to carbohydrate binding by moDCs; fucose could be preferentially internalised through MR (not inhibited by HMW biofilm carbohydrates) and Lewis^X^ through DC-SIGN and/or MR (both inhibited by HMW biofilm carbohydrates).

Next, to establish whether biofilm carbohydrates modulated other aspects of moDCs biology, we investigated their ability to induce morphological changes (Fig. 6b) by plating moDCs on surfaces coated with HMW-2 (prior to LPS removal) or LPS-free HMW-2. MoDCs cultured with LPS-free HMW-2 for 24 h displayed a rounder shape, based on their increased Circularity Index, and reduced Perimeter (Fig. 6b). These morphological changes were less apparent when using LPS-containing HMW-2 indicating that LPS could partially reverse this effect. These findings are consistent with previous results showing reduced DC dendrite formation after DC-SIGN engagement in moDCs^48^.

We also explored whether HMW-2 could affect DC-SIGN surface expression in these assays (Supplementary Fig. 16). Total DC-SIGN surface expression (Raw Integrated Density) was reduced by HMW-2 (both crude and LPS-free preparations), but when adjusting for cell perimeter (Signal per Unit Area) only the cells exposed to LPS-containing HMW-2 displayed reduced surface DC-SIGN, which is compatible with a more classical moDC activation caused by LPS (23).

We next determined the effect of LPS-free HMW-2 (10, 1 and 0.1 µg/ml) on cytokine production by moDCs. LPS-free HMW-2 did not induce cytokine release by moDCs nor affect cytokine responses to *E. coli* LPS (100 and 10 ng/ml) at 4 h (Fig. 6c). We also investigated whether addition of LPS-free HMW-2 could affect the cytokine response of moDCs to fixed biofilms and planktonic cultures using the Δ*wspF* Δ*psl* (biofilm-producing) or Δ*wspF* Δ*psl* Δ*pel* (biofilm-deficient) mutant strains, respectively (Supplementary Fig. 17). Results show similar production of TNF-α, IL-6, Gro-α, IL-23 and IL-10 in response to Δ*wspF* Δ*psl* biofilms and Δ*wspF* Δ*psl* Δ*pel* cultures and increased production of IL-1β in response to Δ*wspF* Δ*psl* biofilms compared to Δ*wspF* Δ*psl* Δ*pel* cultures (p < 0.01). As above, LPS-free HMW-2 did not induce cytokine production by moDCs nor affect cytokine responses to both types of *P. aeruginosa* cultures, which would be in agreement with their ability to bind DC-SIGN (Fig. 1). Combined these findings support a role for biofilm carbohydrates in modifying moDC biology beyond modulation of cytokine production in response to bacterial agonists.

## Discussion

**Fig. 7:**
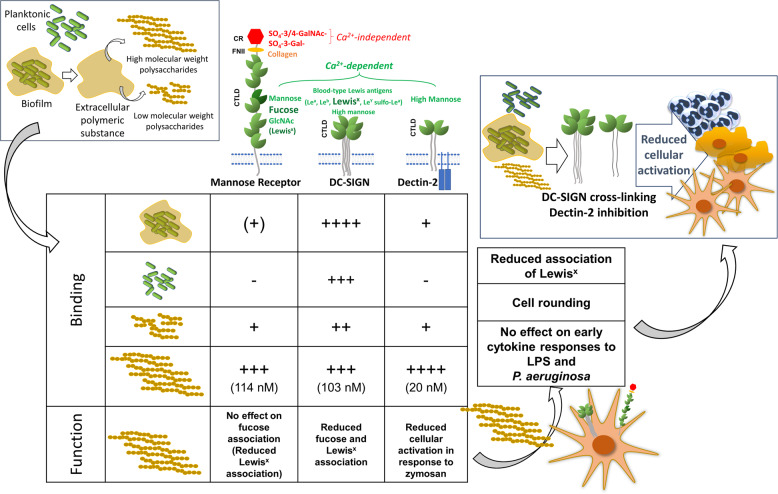
Involvement of C-type lectin receptors in the recognition of P. aeruginosa. *P. aeruginosa* biofilms, planktonic cells and high and low molecular weight biofilm carbohydrates were tested for their interaction with three important C-type lectins: DC-SIGN, MR and Dectin-2. DC-SIGN seems to play a dominant role in *P. aeruginosa* recognition based on its ability to bind biofilms, planktonic cells and purified biofilm carbohydrates, while Dectin-2 and MR preferentially bind purified biofilm carbohydrates. Functionally, purified biofilm carbohydrates interfere with DC-SIGN, Dectin-2 and, partially, MR activities and influence the phenotype of immune cells (moDCs). These findings implicate C-type lectins, particularly DC-SIGN, and to some extent Dectin-2, in the detection of *P. aeruginosa* infection and support a potential role for biofilm carbohydrates in modulating immunity to *P. aeruginosa.* DC-SIGN engagement and Dectin-2 blocking in a variety of innate immune cells such as DCs, macrophages and neutrophils could lead to immune evasion by reducing cellular activation.

Several new findings have emerged from this study. We (i) demonstrated robust recognition of *P. aeruginosa* biofilms and planktonic cells by DC-SIGN (CD209), and weak recognition of biofilms by MR (CD206) and Dectin-2; (ii) showed binding of DC-SIGN, MR and Dectin-2 to purified biofilm carbohydrates, particularly to the high molecular weight fractions; (iii) provided evidence for biofilm carbohydrates affecting DC-SIGN, MR and Dectin-2 function and (iv) established the ability of biofilm carbohydrates to modulate moDC phenotype. The key message of these studies is that *P. aeruginosa* produces carbohydrates that have the potential to influence immunity through the engagement of C-type lectins (Fig. 7). Only MR has been previously implicated in the recognition of *P. aeruginosa* biofilms; MR alongside TLR-2, contributed to the response to Slime-GLP, a crude ethanol extract of *P. aeruginosa* biofilm matrix^49^.

Biofilm-binding assays strongly support a dominant role for DC-SIGN in recognition of *P. aeruginosa* biofilms compared to MR and Dectin-2. This enhanced binding could relate to increased recognition of the biofilm matrix as suggested by our confocal study; while ligands for all three lectins appeared to concentrate into clusters, additional DC-SIGN ligands were more widely distributed and included material dispersed among bacterial aggregates. In addition, although not apparent in the confocal images, there is a possibility of DC-SIGN recognising bacterial cells within biofilms as these cells tend to retain CPA LPS expression^50^ which is required for DC-SIGN binding to planktonic cells. Despite the clear differences between DC-SIGN, MR and Dectin-2 in their biofilm-binding capabilities, their similar interaction with purified biofilm polysaccharides in two independent experimental settings (lectin ELISAs/Fc proteins and BLI/full-length extracellular regions) suggests that biofilms can generate ligands for DC-SIGN, MR and Dectin-2 and that steric hindrance could impair access to Dectin-2 and MR ligands within biofilm structures, potentially because of the presence of carbohydrate-binding proteins such as CdrA^51^ and/or LecB^52,53^, which could share binding sites with MR and/or Dectin-2 ligands. Additionally, since binding to whole biofilms in the lectin ELISA assays was tested after extensive washing, it is possible that “firmly associated” (washing-resistant) and “loosely associated/secreted” (removed by washing) biofilm carbohydrates are differentially recognised by DC-SIGN, MR and Dectin-2. DC-SIGN could bind to both “firmly associated” and “loosely associated/secreted” biofilm carbohydrates, while MR and Dectin-2 might preferentially bind to the “loosely associated/secreted” fraction. Culture conditions could also play a role as exemplified by our confocal analysis when Dectin-2 bound substantially better than MR to biofilms grown underflow conditions while both displayed similar binding to biofilms grown statically in 96-well plates.

Our data do not support a requirement for the *psl* operon, responsible for the production of the neutral mannose-rich carbohydrate Psl^8,9^, for the biosynthesis of DC-SIGN, MR and Dectin-2 ligands (Fig. 1). In particular, DC-SIGN binds to biofilms and planktonic cells regardless of the presence of Psl or Pel. In relation to these findings, the predicted structure for our HMW polysaccharide preparations differs from the repeating pentameric units of D-mannose, L-rhamnose and D-glucose previously proposed for Psl^9^. Both HMW exopolysaccharide preparations contain a small proportion of galactose and lack mannose β anomers. There is a high proportion of 1-6-linked-α-mannose with some 1-2 linkages, which is characteristic of mannans (Supplementary Fig. 7a). In *Candida albicans* the structure of mannan varies depending on culture conditions^54^ and it is highly feasible that differences in growth conditions (which among other things can affect the expression of CdrA and LecB^52^, purification procedures and bacterial strain employed (WT vs Δ*wspF* Δ*pel*) could account for these observations. Of note, the structure described by Byrd et al.^9^ is for one of two LMW carbohydrate fractions obtained by Sephadex G50 chromatography. Both LMW fractions were diminished when Psl was absent but were not recognised by an anti-Psl antiserum^9^, that preferentially bound the HMW carbohydrate fraction in a *psl*-dependent manner. Intriguingly, the lack of Psl in this experimental setting did not lead to a major loss of material in the HMW carbohydrate fraction^9^ which suggests that under the experimental conditions used by the authors there could be a substantial amount of HMW exopolysaccharides in *P. aeruginosa* biofilms produced independently of the *psl* operon.

The strain Δ*wspF* Δ*pel*^38^ used to generate the carbohydrates in this study produces high cyclic-di-GMP levels that could impact on the regulation of carbohydrates. For instance, McCarthy et al. demonstrated regulation of LPS modifications by cyclic-di-GMP in *P. aeruginosa* through binding to WarA, a methyltransferase that regulates CPA LPS modal distribution^55^. Exopolysaccharides from Δ*wspF* Δ*pel* are recognised by the Psl/mannan-binding lectin LecB which fails to bind a similar preparation from an isogenic Δ*wspF* Δ*psl* strain^52^. These results indicate that the synthesis of at least a proportion of the mannose-rich sugars within the Δ*wspF* Δ*pel* material depends on the *psl* operon. Currently, we do not know the contribution of the *psl* operon to the production of the HMW polysaccharides characterised in this study, nor if it facilitates generation of DC-SIGN (and MR and Dectin-2) ligands within these preparations. To address these questions, work is in progress to purify and analyse carbohydrates from PAO1, Δ*wspF* Δ*psl* and Δ*wspF* biofilms and study their recognition by C-type lectins. In the case of MR and Dectin-2, there was some indication of preferential binding of both lectins to Psl-containing biofilms (Fig. 1).

In agreement with our carbohydrate analysis, Bates et al. using the same purification procedure as ours^42^ identified galactose alongside mannose, glucose and rhamnose in carbohydrates generated from biofilms produced by two *P. aeruginosa isolates* (700829 and 700888) and there was an abundance of (2–6) linked (32–28%), 2-linked (20–19%), 3-linked (16%), and terminal (23–27%) mannose. Hence, there is an exciting possibility for mannose-rich carbohydrates in *P. aeruginosa* not being restricted to Psl, and/or not conforming to a unique structure but displaying adaptability to environmental changes and/or bacterial genetic makeup further broadening the range of biofilm architecture and associated immune responses. The presence of galactose in Psl preparations has been observed previously^56^.

It is intriguing that CPA LPS is required for the binding of DC-SIGN to planktonic cells. The CPA O-antigen consists of repeating units of [-3)D-rhamnose(α1-3)D-rhamnose(α1-2)D-rhamnose(α1-] and does not conform to the expected structure of DC-SIGN ligands^33^. We detected binding of DC-SIGN to LPS purified from *P. aeruginosa* serotype 10.2, strain ATCC 27316, using lectin ELISA (data not shown), which would be consistent with direct recognition of CPA LPS by DC-SIGN, but at this stage, we cannot rule out binding of DC-SIGN to the OSA component produced by this serotype or to contaminating carbohydrates. CPA LPS has been implicated in biofilm maturation^41^ and it would be of interest to establish whether this could be linked to the generation of DC-SIGN-binding carbohydrates that require CPA LPS for their secretion and/or synthesis. This hypothesis would be consistent with the lack of matrix materials in the CPA^−^/OSA+ (Δ*rmd*) biofilms described by Murphy et al.^41^. The *rmd* gene product is responsible for synthesis of D-rhamnose and does not contribute to the biosynthesis of any other carbohydrate structures such as OSA, alginate or rhamnolipids^57^.

Based on our findings it is plausible to speculate that following initial infection by planktonic cells, the biofilm lifestyle could promote chronicity not only by protecting against the immune attack and antibiotic treatment but also by influencing immune activation through the engagement of lectin receptors by biofilm carbohydrates. The consequences of these interactions will strongly depend on the cellular background, co-engagement of other receptors and ligand valency^58^. Our functional studies support interference with DC-SIGN and Dectin-2 activities by biofilm carbohydrates that could plausibly affect the biology of immune cells expressing these lectins. A major effect of biofilm carbohydrates on MR activity appears less likely. In the case of moDCs (DC-SIGN^+^/MR^+^/Dectin-2^low/negative^), purified biofilm carbohydrates reduced Lewis^x^ uptake and increased circularity. If these effects are due to DC-SIGN engagement, biofilm carbohydrates could interfere with DC-T cell interactions or DC migration through inhibition of DC-SIGN binding to ICAM-3 or ICAM-2, respectively^59,60^. Early work in moDCs described a unique signalling pathway triggered by DC-SIGN leading to activation of Rho-GTPases and an immature DC phenotype with reduced dendrite formation and ability to induce T cell proliferation^48^. Future work will centre on the ability of biofilm carbohydrates to induce Rho-GTPases activation and the consequences of this interaction on the capacity of moDC to activate T cells. DC-SIGN is strategically placed to sense *P. aeruginosa* infection. Previous observations linked DC-SIGN expression in DCs with biofilm positivity in chronic rhinosinusitis with nasal polyposis^61^, and suggest unique immune responses in the presence of biofilms that correlate with DC-SIGN expression. In human skin, dermal macrophages express DC-SIGN^62^ and could contribute to immune responses to *P. aeruginosa* wound infections. DC-SIGN expression in alveolar macrophages has been described during infection in tuberculosis patients^63^.

Dectin-2 belongs to a group of activating C-type lectin receptors and we initially envisaged its engagement by biofilms as a counterbalance to immune evasion promoted by DC-SIGN and MR. Also, while DC-SIGN expression occurs in tissue macrophages and DCs and could contribute to initial detection of infection, Dectin-2 could be involved after activation and recruitment of inflammatory monocytes^64,65^. Work with Dectin-2 reporter cells indicate that biofilm carbohydrates could hinder Dectin-2-mediated cellular activation, which could act as an additional immune evasion mechanism provided by biofilms.

Mouse models offer an excellent setting to investigate cellular collaborations in response to infection. This is particularly important in the case of C-type lectins whose expression is cell- and activation state-dependent. As an example, the collaborative contribution of C-type lectins expressed by migratory monocytes during *C. albicans* infection was recently characterised using double and triple knock outs for Dectin-1, Dectin-2 and Mincle^66^. Since DC-SIGN appears to have a dominant role in *P. aeruginosa* recognition, experimental models need to consider lack of DC-SIGN orthologs in mice^15^. Researchers have addressed this issue through the use of DC-SIGN transgenic mice^67,68^ and we propose that any analysis of the contribution of C-type lectins to *P. aeruginosa* infections, should be performed in the context of DC-SIGN expression using DC-SIGN transgenic animals. Finally, while this work has focused on how biofilm carbohydrates could impact bacterial infection, it is important to consider that polymicrobial communities are frequently found during chronic infections^69^. Since bacteria and fungi co-exist in human diseases, we predict that biofilm carbohydrates could have a major impact on the recognition of fungal pathogens by the host. In summary, this work demonstrates direct interaction between different C-type lectins and carbohydrates generated by *P. aeruginosa* as planktonic cells and biofilms and highlights the possibility that these receptors contribute to immune responses during *P. aeruginosa* infection.

## Methods

### Biofilm quantification assay

All strains (Table 1), unless otherwise stated, were grown on Lysogenic Broth (LB) agar plates from glycerol stocks stored at −80 °C and incubated overnight at 37 °C. Overnight cultures in X-Vivo-15 medium (Lonza) (5 ml, 37 °C, 200/220 rpm) diluted to OD_600nm_ 0.01 were cultured for 3 h at 37 °C, 200/220 rpm. The OD_600nm_ of mid-log phase cultures in X-Vivo-15 was adjusted to 0.04 OD_600nm_ and 100 μl of cultures were added into each well of a UV-sterilised 96-well plate [Costar (9017, Corning) or Maxisorp (439454, Nunc immune-plate)]. Cultures incubated for 24 h at 37 °C, 5% CO_2_ were washed three times with 200 μl of HPLC water and stained with 125 μl of 1% (w/v) crystal violet (1 h, room temperature (RT)). After washing three times in water, the stain was solubilised by adding 200 μl of 70% ethanol for 15 min; 125 µl was transferred into a clean 96-well Costar plate to measure the absorbance at 595 nm using a Multiskan FC (Thermo Scientific).

### Analysis of the adhesion of planktonic *P. aeruginosa* to plastic

Overnight *P. aeruginosa* cultures were centrifuged at 16,000 × g for 5 min at 4 °C, washed twice with PBS, and re-suspended in 4% paraformaldehyde (PFA, 15710-S, Electronic Microscopy Sciences, USA) in PBS for 30 min at 4 °C. After fixation, cultures were washed once with PBS, adjusted to 0.5 OD_600nm_ in PBS, and pipetted onto Maxisorp plates (50 µl/well). After washing three times with PBS wells were blocked by adding 3% (w/v) bovine serum albumin (BSA) (80400-100, Alpha diagnostics, 50 µl/well) in PBS and then incubated with rabbit anti-*P. aeruginosa* polyclonal antibody (50 µl/well, ab68538, Abcam) diluted 1:1000 in PBS for 90 min at RT. After three washes in PBS, the plate was incubated with goat anti-rabbit IgG conjugated to alkaline phosphatase diluted 1:2000 (A3687, Sigma, 50 µl/well) in PBS for 1 h at RT. After three washes with AP buffer (100 mM Tris-HCl, 100 mM NaCl, 1 mM MgCl_2_, pH 9.5), 50 µl of p-nitrophenyl phosphate substrate solution (Sigma) were added to each well and incubated for 30-40 min at room temperature in the dark. Absorbance was measured at 405 nm using a Multiskan FC (Thermo Scientific).

### Lectin binding assays

Assays for the binding of Fc-chimeric proteins to fixed *P. aeruginosa* biofilms, fixed planktonic *P. aeruginosa* cells and purified carbohydrate were performed as follows. Biofilms were grown on a Maxisorp (439454, Nunc immune-plate) plate over 24 h and fixed with 50 µl of 2% PFA in PBS for 10 min at 4 °C. For planktonic *P. aeruginosa* cells, wells of Maxisorp plates were coated with fixed bacteria (100 µl/per well) and incubated at 4 °C overnight. Purified biofilm carbohydrate was added to Maxisorp plates overnight (50 µl/well in 154 mM NaCl, 37 °C). In all instances, plates were washed three times with TSB (10 mM Tris-HCl, pH 7.5, 10 mM CaCl_2_, 154 mM NaCl and 0.05% (v/v) Tween 20). Chimeric proteins MR-CTLD4-7 (CTLD4-7-Fc, prepared in house,^35^), DC-SIGN (DC-SIGN-Fc, R&D Systems) and Dectin-2 (Dectin-2-Fc, Enzo) (50 µl/well in TSB) were added and incubated for 2 h at RT. After three washes with TSB, antihuman Fc-conjugated to alkaline phosphatase (A9544, Sigma) was added (50 µl/well) and incubated for 1 h, RT (1:1000 dilution). After washing three times with TSB, alkaline phosphatase activity was measured as above. Inhibition assays were carried out as above but using TSB buffer containing 1 M NaCl (TSB-high salt). DC-SIGN was preincubated with different concentrations of the monosaccharides: mannose (63579, Fluka), fucose (47870, Fluka), or galactose (4829, Fluka) in TSB-high salt for 30 min at RT. Dectin-2 (and MR-CTLD4-7) was incubated with different doses of Manα1-2Man (M202, Dextra) under the same conditions. After pre-incubation, proteins were added to appropriate wells containing biofilms. Polymers containing L-fucose, Lewis^x^ or D-galactose (2-5 µg/ml, 50 µl per well, Lectinity) and heat-killed *C. albicans* were used as controls. To test the ability of biofilm carbohydrates to inhibit binding of DC-SIGN and MR to fucose-PAA and binding of Dectin-2 to zymosan, wells were coated with L-fucose-PAA (2 µg/ml) or zymosan 1 × 10^6^ particles per well in 50 µl and proteins were preincubated with different concentration of HMW-1 in TSB for 1 h and added to wells. Binding was detected as above.

### Study of DC-SIGN, MR-CTLD4-7, and Dectin-2 binding to *P. aeruginosa* biofilms by confocal microscopy

Biofilms were generated underflow on µ-Slide VI 0.4 (Ibidi), as follows: Mid-log phase cultures in X-Vivo 15 medium (pre-gassed for 48 h at 37 °C / 5% CO_2_) were diluted to OD_600nm_ 0.04 and used to inoculate μ-Slide VI 0.4 channels (30 µl per channel, tissue culture treated) and incubated at 37 °C for 1 h. Slides were fitted into a syringe pump system, and exposed to flow (1.6 µl/h, pre-gassed X-Vivo-15) at 37 °C, 5% CO_2_. After culture, supernatants were collected, biofilms fixed with 100 µl 4% PFA in PBS for 10 min at 4 °C and washed three times with TSB buffer. Wells were stained with FM 1-43 FX membrane dye (100 µl per channel, 2–10 µg/ml, F35355, Thermofisher) in PBS for 30 min on ice. Following three washes with TSB buffer MR-CTLD4-7, Dectin-2 or DC-SIGN Fc-chimeric proteins (30 µl per channel, 10 µg/ml in TSB buffer) were added and incubated for 2 h at RT. After 3 washes in TSB buffer, goat anti-Human IgG conjugated to Alexa fluor 647 (10 µg/ml, A21445, Invitrogen) and 3%(v/v) Goat serum (D9023, Sigma) in TSB were added and incubated for 1 h at RT. After 3 washes with TSB, DNA was labelled with DAPI (2 µg/ml, D9542, Sigma-Aldrich) in PBS for 15 min, RT. Plates were washed with TSB and mounted in Ibidi mounting media (50001, Ibidi) before storing at 4 °C in the dark. Confocal images were acquired using Zeiss LSM 880 using a 40x/1.20 water objective, the collection was not done with filters. Fluorescence emission was collected between 434 and 515 nm (DAPI), 469-538 nm (FM 1-43FX), 641-688 nm (AF 647). Stack size (49.43 µm, y: 49.43 µm, z: 7.5–12.9 µm). Images were processed using Fiji^70^.

### Carbohydrate purification

Carbohydrate was extracted^42^ from cultures of ∆*wspF* ∆*pel*^7^ in TSB medium in 1.5 L flasks (400 ml per flask) and incubated statically at 37 °C for five days. Cultures were treated with 0.02% formaldehyde (v/v) (33220, Sigma-Aldrich) (1 h, RT, 100 rpm) followed by 275 mM NaOH (3 h, RT, 100 rpm, S318-1, Fisher Scientific) and centrifuged (16,000 × g, 1 h, 4 °C). Supernatants were collected, filtered and dialysed/concentrated against HPLC water using VIVAFLOW 200, 12–14 kDa MWCO membrane (Sartorius Stedim Biotech) to a maximum final volume of 100 ml. Concentrates were treated with tri-chloro-acetic acid (TCA, 20% (w/v), 3000-50, Fisher scientific) at 4 °C for 30 min, centrifuged (16,000 × g, 1 h at 4 °C) and supernatant incubated with 1.5 volumes of cold 95% (v/v) ethanol (−20 °C, 24 h). The solution was centrifuged at 16,000 × g for 1 h at 4 °C and the pellet was re-suspended in HPLC water. This step was done twice to improve purity. The solution was then dialysed against HPLC water using 8–10 kDa MWCO Spectra/Por Float-A-Lyzer G2 Dialysis Device (Z726508, Sigma-Aldrich), and lyophilized. The lyophilized powder was re-suspended in PBS (pH 7.4) and fractionated on a HiPrep 26/60, Sephacryl S-200 HR gel filtration column (17119501, GE Healthcare) calibrated with protein standards (1511901, Bio-rad). For the gel filtration, the equivalent of 2.4 or 3.6 litres of culture were pooled for each column run. High molecular weight (>45 kDa) and low molecular weight preparations (<45 kDa) were pooled, dialysed against HPLC water and lyophilized. Endotoxin was eliminated by repeated passages (x10) through Pierce high capacity endotoxin removal spin columns (88274, Thermofisher) following the manufacturer’s recommendations.

### Carbohydrate analysis

#### Molecular weight analysis

Molecular weight, polydispersity and polymer distribution values were derived from gel permeation chromatography (GPC) with a Viscotek/Malvern GPC system consisting of a GPCMax autoinjector fitted to a TDA 305 detector (Viscotek/Malvern, Houston, TX). The TDA contains a refractive index detector, a low angle laser light scattering detector, a right-angle laser light scattering detector, an intrinsic viscosity detector and a UV detector (λ = 254 nm). Three Waters Ultrahydrogel columns, i.e. 1200, 500 and 120, were fitted in series (Waters Corp. Milford, MA). The columns and detectors were maintained at 40 °C within the TDA 305. The system was calibrated using Malvern pullulan and dextran standards. The mobile phase was 50 mM sodium nitrite. The mobile phase was filter sterilized (0.45 µm) into a 5 L mobile phase reservoir. Biofilm carbohydrates samples were dissolved (6 mg/ml) in mobile phase (50 mM sodium nitrite, pH 7.3). The samples were incubated for ~60 min at 60 °C, followed by sterile filtration (0.45 μm) and injected into the GPC (50–200 μL). Sample recovery was routinely >90%. Dn/dc for each sample was calculated using the OmniSec software (v. 4.6.1.354). The data were analysed using a single peak assignment in order to obtain an average molecular weight for the entire polymer distribution. Each sample was analysed in duplicate or triplicate. Replicate analysis of calibration standards indicated reproducibility of +/−3%, which is well within the limits of the technique.

#### Proton nuclear magnetic resonance

Carbohydrate preparations were structurally characterised by solution-state 1D ^1^H NMR spectroscopy and 2D COSY and HSQC NMR spectroscopy. 1D NMR data acquisition and analysis were based on methods from Lowman et al.^43,54^. Briefly, NMR spectra were collected on a Bruker Avance III 400 NMR spectrometer operating at 331°K (58 °C) in 5 mm NMR tubes. Each carbohydrate (about 5 mg) was dissolved in about 550 µl dH_2_O (Cambridge Isotope Laboratories, 99.8 +% deuterated). Chemical shift referencing was accomplished relative to TMSP at 0.0 ppm. The proton 1D NMR spectra were collected with 36 scans, 65,536 data points, 20 ppm sweep width centred at 6.2 ppm, and 1 s pulse delay and processed using exponential apodization with 0.3 Hz line broadening. COSY spectra were collected using 2048 by 128 data points, 16 dummy scans, 64 scans, and 9.0 ppm sweep width centred at 4.5 ppm and processed with sine apodization in both dimensions and zero-filled to 1024 data points in f1. HSQC spectra were collected using 1024 by 256 data points, 4 dummy scans, 128 scans, and 6.0 ppm sweep width in f2 and 185 ppm sweep width in f1 and processed with qsine apodization in both dimensions and zero-filled to 1024 data points in both dimensions. Polymer hydrolysis was accomplished by heating the isolate in 4 M TFA at 100 °C for 5 h. Processing was accomplished with Bruker TopSpin (version 4.0.6) on the MacBook Pro.

### Measurement of HMW-2 binding to recombinant human MR, Dectin-2, and biotinylated DC-SIGN by BLI

Binding experiments were performed on an Octet K2 biolayer interferometry system (ForteBio, San Jose, CA) in 10X Kinetics Buffer at 30 °C and 1000 rpm. Black bottomed 96-well plates were from Grenier Bio-One and the optical biosensor probes from ForteBio. Recombinant human MR (CD206) and Dectin-2 with a poly-His-tag were from R&D Systems (Minneapolis, MN). DC-SIGN was purified from cultures of *E. coli* BL21/DE3 transformed with the pT5T vector containing the cDNA encoding for the extracellular region of DC-SIGN. Bacterial pellets from cultures treated with isopropyl-β-d-thiogalactoside were lysed by sonication. The material in inclusion bodies was solubilized using 6 M guanidine-HCl, 10 mM Tris-HCl, pH 7.0, and 0.01% β-mercaptoethanol. Extracts were dialysed against loading buffer (1.25 M NaCl, 25 mM Tris-HCl, pH 7.8, 25 mM CaCl_2_) and DC-SIGN was purified by affinity chromatography using Man-Sepharose and anion exchange chromatography on a Mono Q column (Amersham Biosciences). Biotinylation was performed using N-hydroxysuccinimide biotin (Sigma-Aldrich, Poole, UK) at a molar ratio of 20:1 reagent: protein at pH 8.4, 4 °C for 60 min^18,71^. Ni-NTA or SA biosensor was placed in the instrument, and after an equilibration period of 3 min the biosensor was exposed to either the poly-His-tagged MR or Dectin-2, or the biotinylated DC-SIGN at a concentration of 0.1 µg/mL for 5 min, and then transferred to 10X Kinetics Buffer for 10 min to establish the BLI signal from the immobilized receptor. Following this, a series of eight similar 5 min exposures to 2-fold increasing concentrations (3.125–400 mg/ml) of carbohydrate each followed by 10 min dissociation in 10X Kinetics Buffer were performed. For each exposure, the equilibrium BLI signal was measured 20 s after the switch to Kinetics Buffer and used in the analysis of binding. A parallel biosensor with immobilized receptor but not carbohydrate was placed in 10X Kinetics Buffer and used to control for receptor dissociation during the experiment. The series of BLI signals for each concentration was fit to models of nonspecific linear binding, saturable specific binding, and specific binding plus nonspecific binding with either local variable or shared global values for each parameter. The sequential F-statistic with a *p* < 0.05 was used to select the most appropriate model for each carbohydrate—receptor interaction. Results are reported as mean values with 95% confidence intervals for apparent equilibrium dissociation constant (K_D_), maximum BLI signal (B_MAX_), and nonspecific binding (K_NS_).

### Cell association assay of fluorescent monosaccharide polymers

U937 cells transfected with human DC-SIGN (U937-DC-SIGN) and control U937 cells were obtained from the American Type Culture Collection (ATCC) and grown in suspension in RPMI-1640 medium (R0883, Sigma) containing 10% (v/v) foetal bovine serum (FBS, F9665, Sigma), 2 mM L-glutamine (35050-038, Gibco), 10 mM HEPES buffer (15630-056, Gibco), 1 mM sodium pyruvate (11360-039, Gibco), 4.5 g/L D-glucose (A24940-01, Gibco) and 0.15% (v/v) sodium bicarbonate (S8761, Sigma). U937 cells were harvested by centrifugation at 250 × *g* for 5 min, washed in Opti-MEM serum-free media (Gibco), re-suspended in Opti-MEM, adjusted to 1.25 × 10^6^ cells/ml and plated in 24 well tissue culture plates (250,000 cells/well, Costar) and incubated for 30 min at 37 °C. Fluorescent monosaccharide polymers: Lewis^X^-PAA-FITC, fucose-PAA-FITC or galactose-PAA-FITC (Lectinity) were added to each well (5 μg/ml final concentration) and Opti-MEM to controls. Cultures were incubated for 1 h at 37 °C and then transferred to fluorescence-activated cell sorting (FACS tubes) and 1 ml of FACS Buffer (0.5% (v/v) FBS, 15 mM NaN_3_ in PBS with Ca^2+^ and Mg^2+^ (D8537, Sigma)) was added to each tube. Cells were washed twice in FACS buffer by centrifugation at 300 × g for 5 min and re-suspended in 200 μl of FACS buffer and fixed with 2% (v/v) PFA in PBS. Cells were analysed using a Beckman Coulter FC500 flow cytometer. Data were analysed using Kaluza analysis software 1.5a. For cell association inhibition assays, cells were preincubated with HMW or Opti-MEM for 1 h at 37 °C before addition of fluorescent monosaccharide polymers. A similar procedure was used for CHO and CHO-MR cells but, being adherent cells, all washes were done in the wells and cells were harvested using trypsin-EDTA for analysis by flow cytometry^45^.

### Effect of biofilm carbohydrate on Dectin-2-mediated cellular activation

Dectin-2 NFκB SEAP reporter cells (HEK-blue 293 muDectin-2, Invivogen) and control HEK cells (Null 1 V, Invivogen) were added to a 96-well plate at 5 × 10^4^ cells per well. To assess Dectin-2 mediated responses, the cells were exposed to different stimuli, i.e. LPS (1 and 10 µg/ml), Zymosan (1 and 100 µg/ml), laminarin (100 and 1 µg/ml) and LPS-free HMW-2 (0.1, 1, 10 and 100 µg/ml) for 18 h. HEK-blue developing reagent were added to wells and plates were read at OD_645 nm_. To analyse the effect of biofilm carbohydrates on the response of Dectin-2 reporter cells to zymosan, 20 µl of LPS-free HMW-2 (0.3 µg/ml), along with negative controls (media, endotoxin free water and PBS) or a positive control (zymosan, 1 µg/ml) were dispensed into a 96-well flat-bottomed plate. Cells (5 × 10^4^ cells in 180 µl HEK-Blue Detection Media per well) were added and incubated for 1 h, 37 °C, 5% CO_2_. Then serial dilutions of zymosan in 20 µl) were added to the competition wells containing the biofilm carbohydrate. The plate was then incubated overnight and read as above. Estimation of the dissociation constant for zymosan followed the method of Furchgott (1966) as adapted by Parker and Waud (1971) for nonlinear analysis^72,73^. Briefly, concentration-response relationships were established for zymosan in the absence of LPS-free HMW-2. Then, individual responses to zymosan in the presence of LPS-free HMW-2 were compared to establish pairs of zymosan concentrations producing equivalent responses in the presence (x) and absence (y) of LPS-free HMW-2. These paired concentrations were fit to a hyperbolic relationship y = (M*x)/K + x) to estimate K_D_ (as K-M). The experiment was repeated 3 times.

### Generation of human monocyte-derived dendritic cells

Human moDCs were prepared from buffy coats (Blood Transfusion Service, Sheffield, UK). Use of buffy coats was approved by the Faculty of Medicine and Health Sciences Research Ethics Committee. PBMCs were isolated using Histopaque-1077 (H8889, Sigma) and monocytes were isolated using human CD14 MicroBeads (130-050-201, Miltenyi Biotec) following the manufacturer’s protocol. Purified monocytes were cultured in RPMI complete medium [RPMI-1640 (R0883, Sigma), 15% (v/v) human AB serum (H4522, Sigma), 2 mM GlutaMAX (G7513, Sigma), 10 mM HEPES (15630056, Gibco), 50 ng/ml recombinant human granulocyte macrophage colony–stimulating factor (rhGM-CSF) (130-093-865, Miltenyi Biotec), and 50 ng/ml rh interleukin (IL)-4 (130-093-921, Miltenyi Biotec)] and cultured at 37 °C, 5%CO_2_ for 6 days. On Day 3, fresh RPMI complete media containing growth factors was added to each well.

### Analysis of changes in moDC morphology after incubation with HMW

µ-Slide VI 0.4 tissue culture treated channels (80606, Ibidi) were coated with HMW (10 µg/ml, in X-Vivo-15) overnight at 4 °C. After washing, and addition of moDCs (5 × 10^4^ cells per channel in X-Vivo 15 containing GM-CSF and IL-4) cultures were incubated for 24 h at 37 °C, 5% CO_2_. Samples were fixed in 4% PFA (15710-s, EM Grade) in PEM (Cytoskeleton-preserving buffer PIPES-EGTA-Magnesium, 80 mM PIPES pH 6.8, 5 mM EGTA, 2 mM MgCl_2_) for 15 min, RT. Slides were washed in PEM and blocked with 5% Donkey serum (D9663, Sigma) in PEM (blocking buffer) in the presence or absence of 0.25% Triton X-100. After 3 washes in PEM, cells were incubated with a rabbit polyclonal antibody against DC-SIGN overnight at 4 °C (10 µg/ml, ab5715, Abcam) in blocking buffer and then with Alexa Fluor 647-AffiniPure Donkey anti-Rabbit IgG (H + L) (711-605-152, Jackson ImmunoResearch) 1:500 in blocking buffer. After 3 washes in PEM, cells were labelled with DAPI (1.5 µg/ml in PBS, D9542, Sigma-Aldrich), washed in PBS and mounted in Ibidi mounting media (50001, Ibidi). Confocal images were acquired using Zeiss LSM 710 under 40x/1.3 oil objective, without filters. Fluorescence emission was collected between 410 and 585 nm (DAPI) and 638–755 nm (AF 647). Image size (212.55 µm, y: 212.55 µm, z: 0.406 µm). Images were analysed using Fiji. Raw Integrated density and cell perimeter were measured by manually following the contour of the cell using the segmented line tool (width = 12 nm). Circularity (the ratio between longer and shortest axes for each cell) was determined by manually following the contour of the cell using the freehand line tool. The selected areas were then saved in the ROI manager for analysis. Single per unit area was calculated by (Raw Integrated density/area selected using the segmented line tool (width = 12 nm) = single per unit area).

### Analysis of moDCs cytokine production in response to *E. coli* LPS in the presence and absence of biofilm carbohydrates

MoDCs were added to 48 well tissue culture plates (10^5^ per well), coated with different doses of LPS-free HMW-2 (10, 1 and 0.1 µg/ml) for 16 h. Cultures were incubated for 2 h and then treated for 4 h with ultrapure *E. coli* LPS (100 and 10 ng/ml, LPS-100, and LPS-10, LPS-EK Ultrapure from *Escherichia coli* Invivogen, cat # tlrl-peklps) and supernatants collected for cytokine quantification. Controls were moDCs incubated only with LPS-free HMW-2. Cytokines were quantified using DuoSet ELISA (R&D Systems) and Magnetic luminex assay kits (R&D Systems).

### Statistical analysis

Statistical analysis was performed in GraphPad Prism. Details of specific tests are provided in figure legends.

### Reporting Summary

Further information on research design is available in the Nature Research Reporting Summary linked to this article.

## Supplementary information

Supplementary Information

Reporting Summary

## Data Availability

Authors can confirm that all relevant data are included in the paper and its supplementary information files.
