# Supplementary material for: Carbohydrates from Pseudomonas aeruginosa biofilms interact with immune C-type lectins and interfere with their receptor function
Source: NPJ Biofilms Microbiomes. 2021 Dec 8;7:87. doi: 10.1038/s41522-021-00257-w (PMC8655052; doi:10.1038/s41522-021-00257-w)
Supplement: Supplementary file 1 — Supplementary Information [file 41522_2021_257_MOESM1_ESM.pdf]

Carbohydrates from *Pseudomonas aeruginosa* biofilms interact with immune C-type lectins and interfere with their receptor function.

Sonali Singh, Yasir Almuhanna, Mohammad Y. Alshahrani, Douglas W. Lowman, Peter J. Rice, Chris Gell, Zuchao Ma, Bridget Graves, Darryl Jackson, Kelly Lee, Rucha Juarez, Janice Koranteng, Sirina Muntaka, Dan Mitchell, Ana C. da Silva, Farah Hussain, Gokhan Yilmaz, Francesca Mastrotto, Yasuhiko Irie, Paul Williams, David L. Williams, Miguel Cámara and Luisa Martinez-Pomares

Supplementary Figures

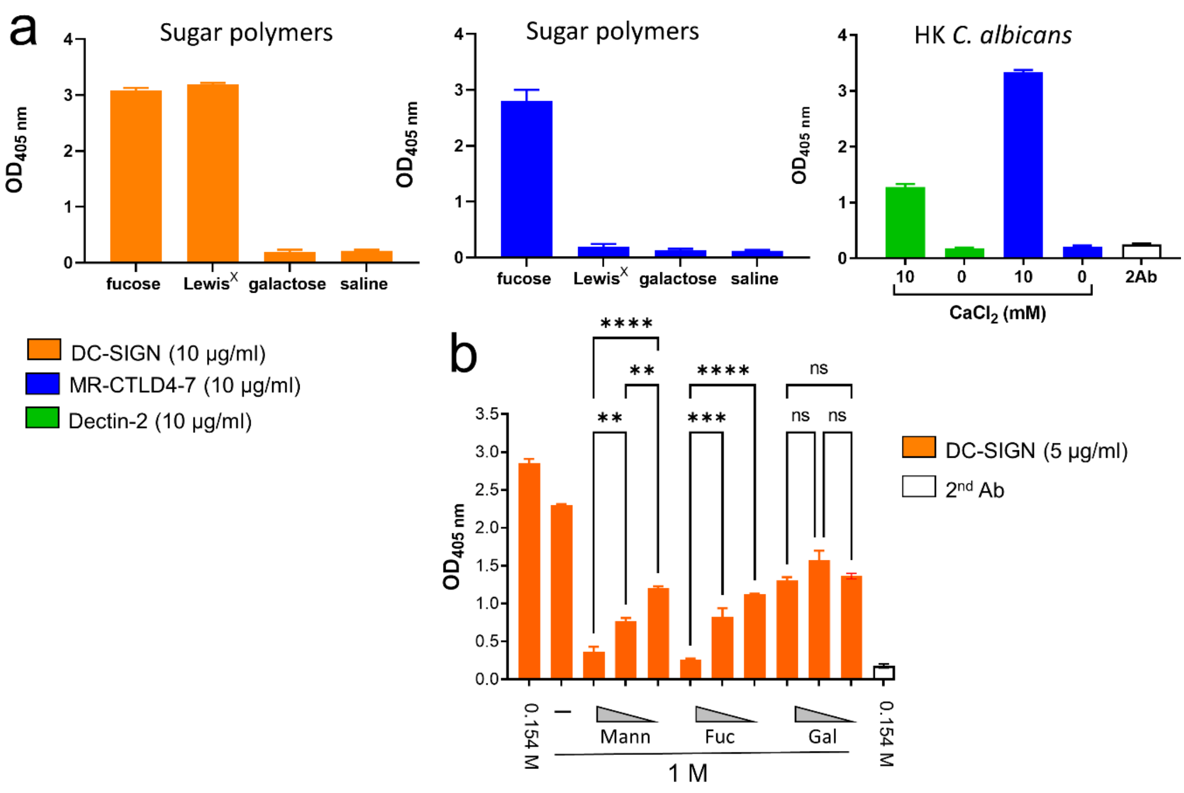

Supplementary Figure 1. Specificity controls for Lectin ELISAs

a. Specificity controls for Lectin ELISAS assays. DC-SIGN preferentially binds to polymers bearing fucose and Lewis^x while MR-CTLD4-7 binds fucose polymers. MR-CTLD4-7 and Dectin-2 bind heat-killed *C. albicans* and binding is Ca^{2+}-dependent.

b. DC-SIGN binding to PAO1 biofilms is selectively inhibited by mannose and fucose compared to galactose. PAO1 biofilms were grown in 96 well plates for 24 h, fixed, then incubated with DC-SIGN-Fc. Assays were performed in two NaCl concentrations (0.154 M and 1 M) and for the 1 M condition, incubation with Fc chimeric proteins was performed in the presence or absence of different concentrations of monosaccharides (1, 0.2 and 0.04 mM). Binding at 1 M NaCl was comparable to that observed at 0.154 M (ns). Presence of monosaccharides significantly reduced protein binding. Mannose and fucose preferentially inhibited binding of DC-SIGN. Man: mannose, Fuc: fucose; Gal: galactose. N=3 in triplicate. One-way ANOVA with Tukey's multiple comparison test.

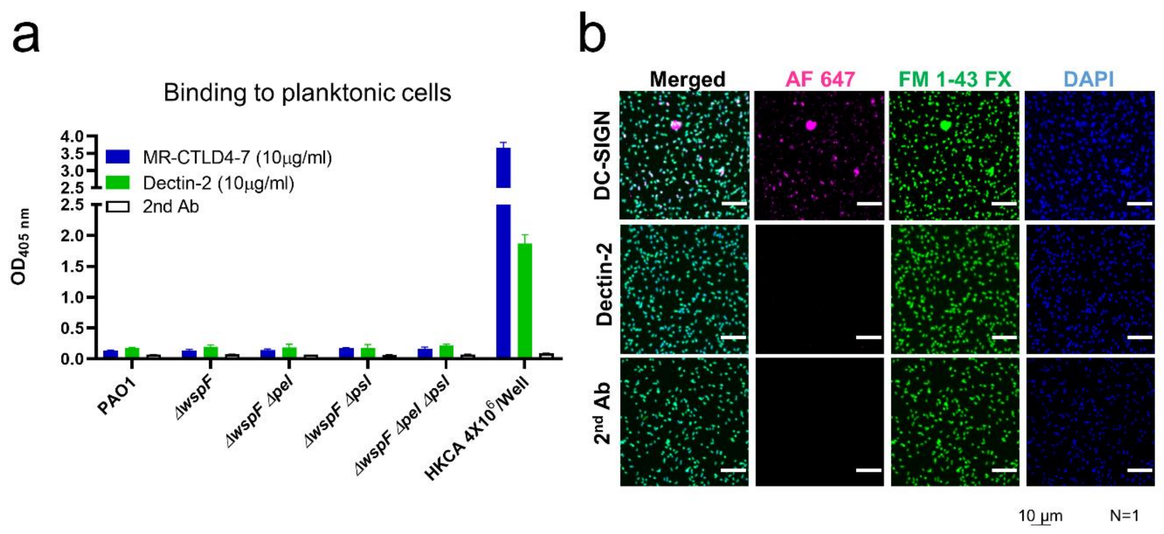

Supplementary Figure 2. Analysis of Dectin-2 binding to planktonic *P. aeruginosa*.

a. Dectin-2 does not bind planktonic *P. aeruginosa* cultures in Lectin-ELISAs. Overnight *P. aeruginosa* cultures were centrifuged at 16,000 × g for 5 min at 4°C, washed twice with PBS, and re-suspended in 4% (v/v) paraformaldehyde in PBS for 30 min at 4°C. After fixation, cultures were washed once with PBS, adjusted to OD_{600nm}=0.5 in PBS, and pipetted onto Maxisorp plates (100 µl/per well) and incubated at 4ºC overnight. Plates were washed three times with TBS (10 mM Tris-HCl, pH 7.5, 10 mM $CaCl_2$, 154 mM NaCl and 0.05% (v/v) Tween 20). Chimeric proteins (50 µl/well in TBS) were added and incubated for 2 h at RT. After three washes with TBS, anti-human Fc-conjugated to alkaline phosphatase was added (50 µl/well) and incubated for 1 h, RT (1:1000 dilution). After washing three times with TBS, alkaline phosphatase activity was measured using p-nitrophenyl phosphate substrate solution (50 µl per well, Sigma). Absorbance was measured at 405 nm using a Multiskan FC (Thermo Scientific). N=2.

b. Dectin-2 does not bind planktonic *P. aeruginosa* cultures in an immunofluorescence assay. Channels of µ-Slide VI 0.4 (Ibidi) slides were coated with fixed *P. aeruginosa* (100 µl per channel, overnight, 4°C), washed three times with TSB buffer, and stained with FM 1-43 FX in PBS for 30 min on ice. Following three washes with TSB buffer Fc proteins (30 µl per channel, 10 µg/ml in TSB buffer) were added and incubated for 2 h at RT. After washing three times with TSB buffer, 100 µl of TSB containing 10 µg/ml goat anti-Human IgG conjugated to Alexa fluor 647 and 3%(v/v) Donkey serum in TBS were added and incubated for 1 h at RT. Following three washes with TSB, the DNA was labelled with DAPI (100 µl per channel, 2 µg/ml) in PBS for 15 min, RT. The plates were washed with TSB and mounted in Ibidi mounting media and stored at 4ºC in the dark. Confocal images were acquired using Zeiss LSM 880 using a 40x /1.20 water objective. Fluorescence emission was collected between 434 and 515 nm (DAPI), 469-538 nm (FM 1-43FX), 641-688 nm (AF 647). In agreement with Lectin ELISAs results, DC-SIGN-Fc bound to planktonic PAO1. N=1.

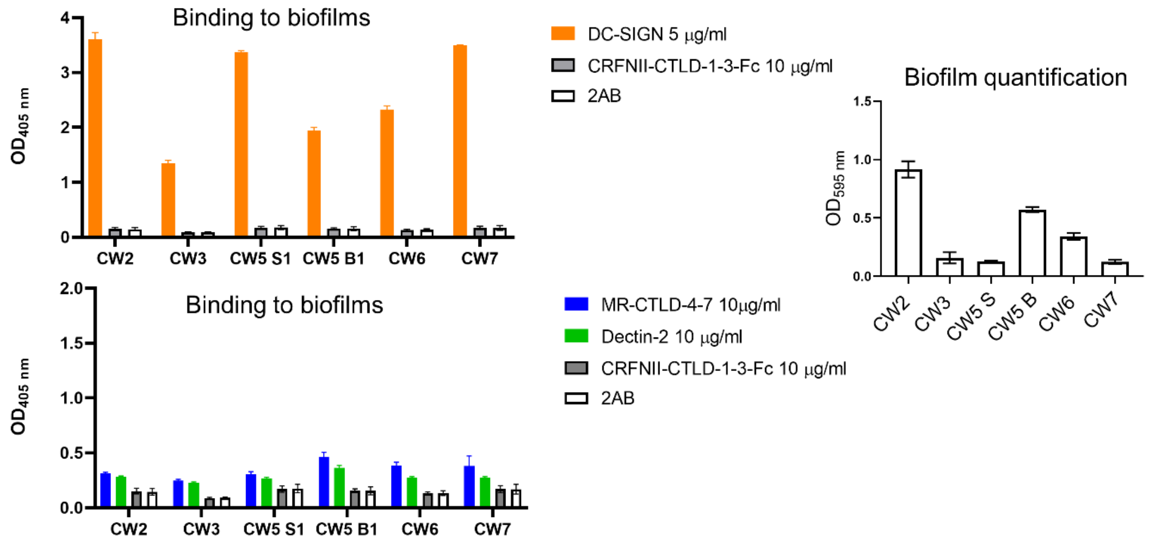

Supplementary Figure 3. Analysis of DC-SIGN, MR and Dectin-2 binding to biofilms formed by *P. aeruginosa* wound isolates.

This strain collection comprises isolates collected from bone (CW2 and CW5-B), wound tissue (CW3, CW6 and CW7) and blood (CW5-S) and two serotypes (CW2, CW3, CW5 and CW6 are serotype O6) and CW7 is part of the serotype O5 cluster, which includes O5, O18, and O20 serotypes. DC-SIGN, MR-CTLD4-7 and Dectin-2 binding follows a similar pattern to that found for laboratory strains, i.e. substantial binding by DC-SIGN compared to MR and Dectin-2. Panel on the right: quantification of biofilm formation by the different isolates using the crystal violet assay.

	CW2	CW3	CW5 B/S	CW6	CW7
**pslA**	-	p.D7E	-	-	-
**pslB**	-	p.V91M	p.V91M	p.G140E	-
**pslC**	p.D173G	p.D173G	p.D173G	p.D173G	p.D173G
**pslD**	p.A108V	-	-	-	-
**pslE**	p.I573V	-	p.I573V	-	-
**pslF**	p.A32S<br>p.A59G<br>p.Y274D	p.A59G<br>p.Y274D	p.A59G<br>p.Y274D	p.A59G<br>p.Y274D	p.A59G<br>p.Y274D
**pslG**	p.L349R	-	-	p.D372N	-
**pslH**	p.T214A	p.T214A	p.T101K<br>p.T214A	p.T214A	p.Q52R<br>p.T214A<br>p.M257B<br>p.S345T
**pslI**	p.D191V<br>p.T222A	p.P314S	-	-	p.T222A
**pslJ**	-	-	-	-	-
**pslK**	p.V213T	-	p.L94F	p.A168V<br>p.V213T	p.V213T<br>p.N424S
**pslL**	p.V289A<br>p.T345A	p.T345A	p.T345A	-	-

Supplementary Figure 4. Sequence analysis confirmed presence of the *psl* operon in all wound isolates analysed in Figure S3 although point mutations could alter the levels and/or structure of the Psl carbohydrate.

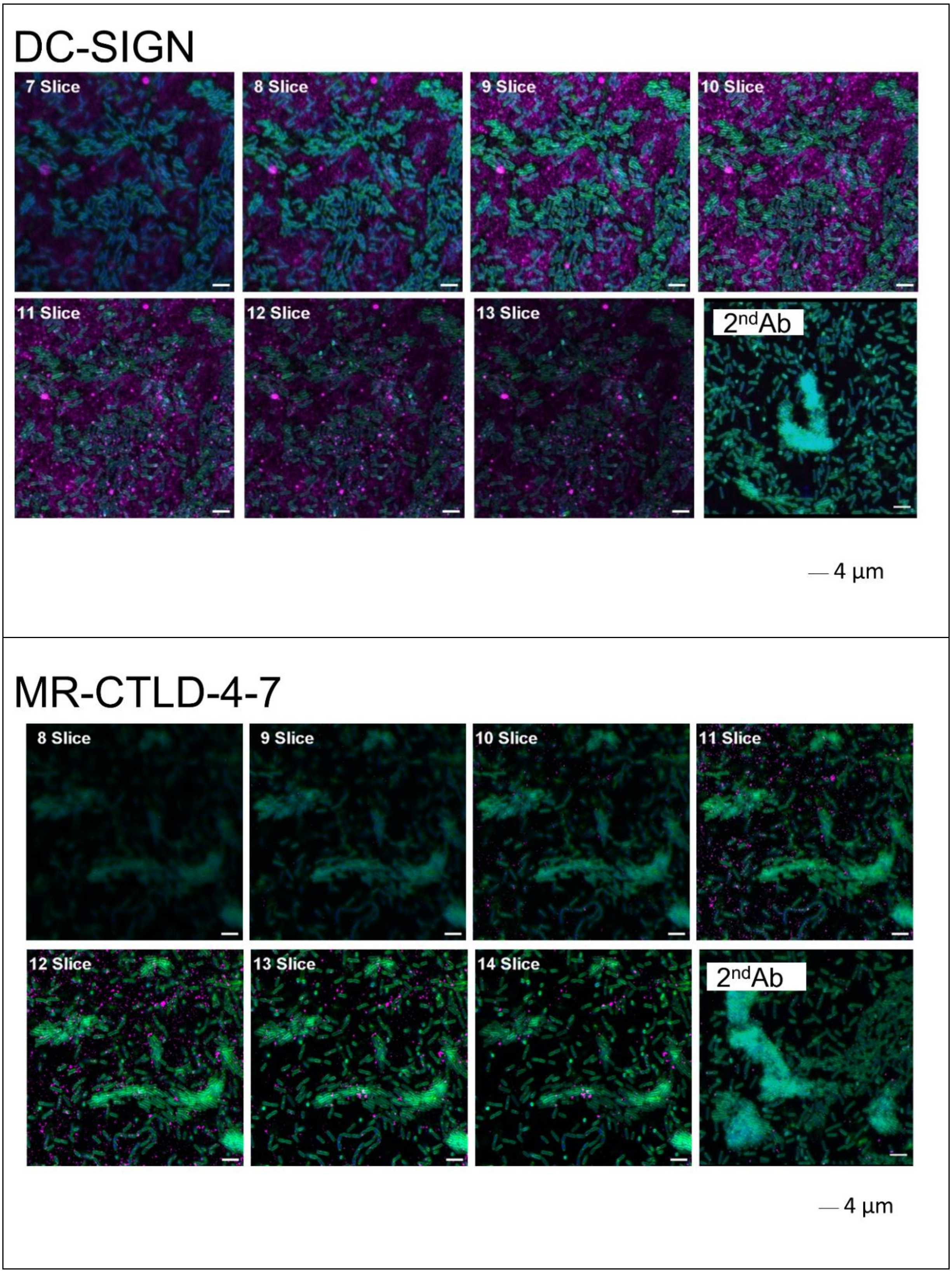
DC-SIGN
7 Slice
8 Slice
9 Slice
10 Slice
11 Slice
12 Slice
13 Slice
2ndAb
4 µm
MR-CTLD-4-7
8 Slice
9 Slice
10 Slice
11 Slice
12 Slice
13 Slice
14 Slice
2ndAb
4 µm

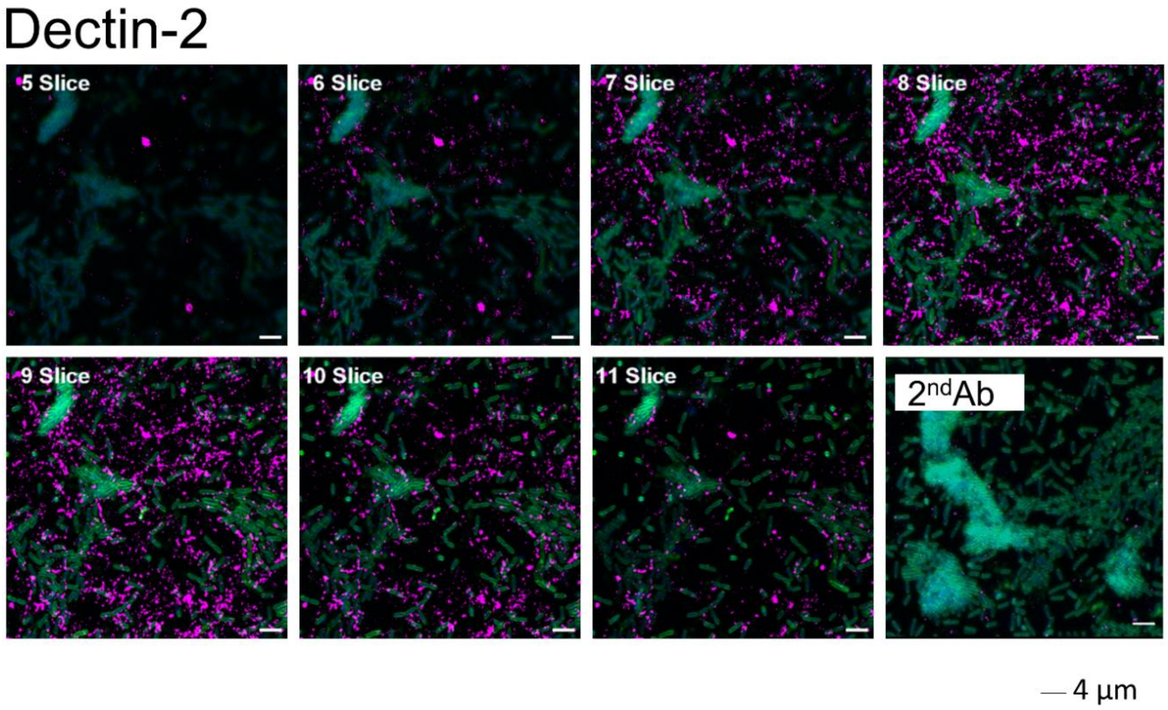

Supplementary Figure 5. Distribution of DC-SIGN, MR and Dectin-2 ligands in *P. aeruginosa* biofilms.

PAO1 biofilms generated under flow conditions for 18 h as described in materials and methods were incubated with DC-SIGN, MR-CTLD4-7 and Dectin-2 Fc-chimeric proteins followed by anti-human Fc-secondary antibody conjugated to Alexa 647 (magenta) and counterstained with DAPI (DNA, blue) and FM1-43FX (bacteria, green). Z-stacks were acquired for all samples using confocal microscopy. Figure show slices from bottom to top covering the bacterial cells for the three receptors and 3D projections of corresponding secondary antibody controls (2nd Ab). Please note that the same control was used for MR and Dectin-2 and has been included in both panels for clarity. The same settings for image acquisition and processing were maintained for test and control samples. Size bar 4 µm. Brightness and contrast settings in Fiji for DC-SIGN and corresponding control 2nd Ab control (AF647 channel): minimum and maximum values 48 and 1497, respectively. For MR and Dectin-2 and 2nd Ab control (AF647 channel): 330 for both. Settings for FM 1-43 FX and DAPI channels were the same in all instances.

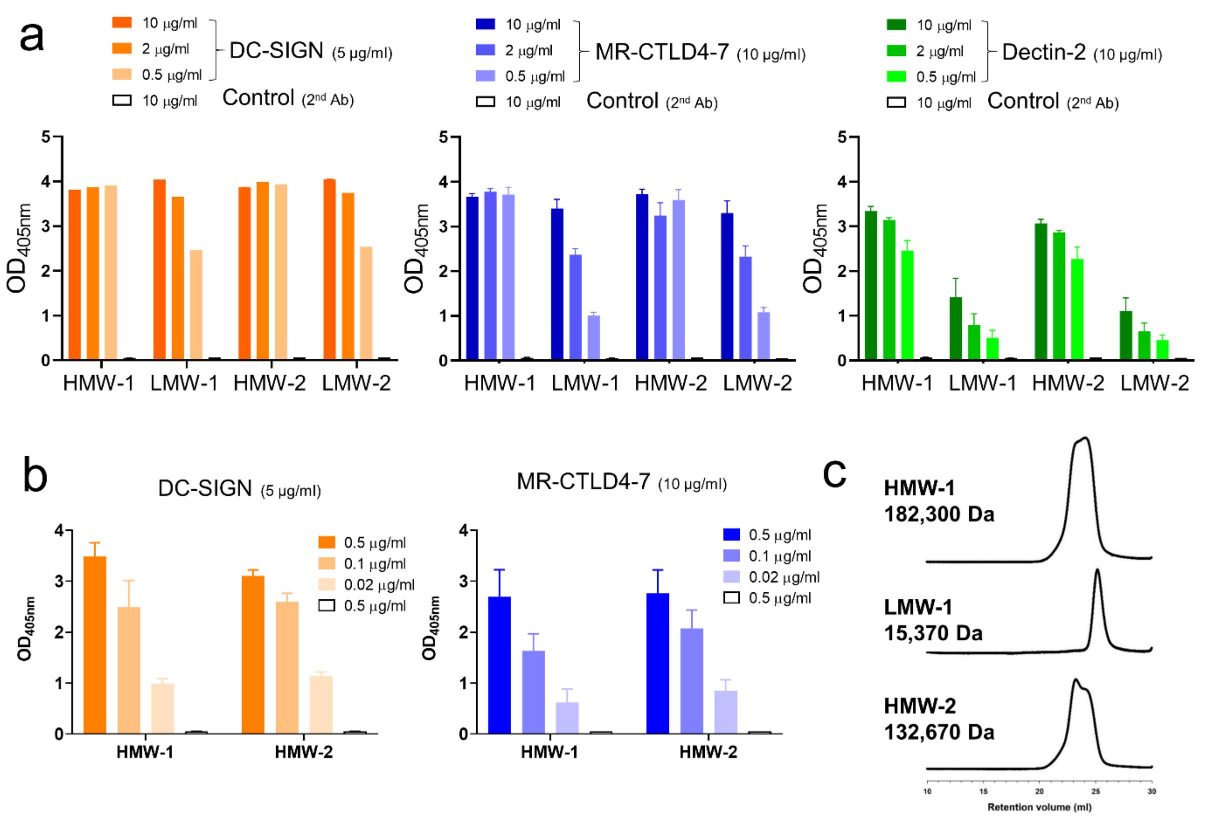

Supplementary Figure 6. Binding of DC-SIGN, MR-CTLD4-7 and Dectin-2 to *P. aeruginosa* biofilm carbohydrates. a. Lectin binding assays demonstrate binding of DC-SIGN, MR-CTLD4-7 and Dectin-2 to high and low molecular weight biofilm carbohydrates (HMW-1, LMW-1, HMW-2 and LMW-2). Robust binding to HMW-1 and 2 was observed at 0.5 µg/ml while binding to LMW-1 and 2 at this concentration was substantially reduced. Fc-chimeric proteins and anti-human Fc-secondary antibody conjugated to alkaline phosphatase were used. Graphs show mean ± SEM of 2 independent repeats done in duplicate. **b.** Further dilution of HMW preparations binding to DC-SIGN-Fc and MR-CTLD4-7-Fc. Graphs show mean ± SEM of 2 independent repeats done in duplicate. **c.** GPC analysis of HMW-1, LMW-1 and HMW-2 confirms successful fractionation into high and low MW forms. HMW-2 contained two peaks poorly resolved. This indicates that this sample is comprised of two components that are similar in MW and perhaps conformation. The MW for HMW-2 reflects the average MW for the entire sample.

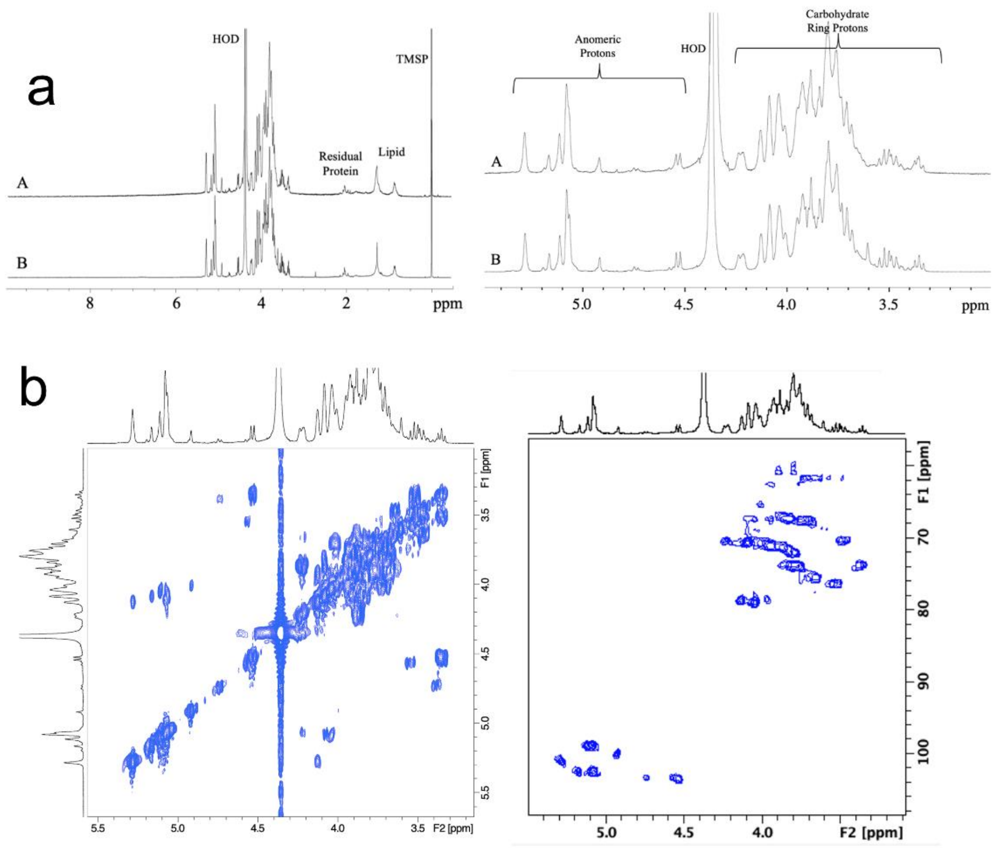

Supplementary Figure 7a: Structural motif characterization of the mannosyl repeat units in HMW-1 (A) and HMW-2 (B) based on ^{1}H NMR.

The 1D proton NMR spectra of HMW-1 and HMW-2 are plotted from 10.0 to -0.5 ppm to show the full NMR spectra and limited impurities in those samples (left). The 1D NMR spectra are expanded between 3.0 and 5.5 ppm to show the details of the carbohydrate spectral region (right). Analysis of the anomeric proton resonances between 4.5 and 5.3 ppm using COSY and HSQC 2D NMR (Figure S7B) provides insight into some of the structural features present. The resonance at 5.12 ppm (C1 at 98.88 ppm) is assigned to an anomeric proton (H1) coupled to the H2 proton resonating at 4.06 ppm (C2 at 71.3 ppm) in a (1-6)-linked mannosyl repeat unit in the backbone with a side chain attached at the 2-position (-6(-2)Mα1-). The resonance at 4.92 ppm (C1 at 99.92 ppm) is assigned to H1 coupled to the H2 proton resonating at 4.01 ppm (C2 at 65.4 ppm) in a (1-6)-linked mannosyl repeat unit in the backbone without a side chain attached at the 2-position (-6Mα1-). The resonance at 5.29 ppm (C1 at 101.11 ppm) is assigned to H1 coupled to the H2 proton resonating at 4.13 ppm (C2 at 74.0 ppm) in a (1-2)-α-linked mannosyl repeat unit in a side chain (-2Mα1-). Side chains may also contain -3Mα1- and -2Mβ1- mannosyl repeat units. Therefore, the mannan segments appear to have a backbone of (1-6)-α-linked mannosyl repeat units connected through their 2-position to short side chains of (1-2)-α-linked mannosyl repeat units on some of the backbone repeat units, but not all of the backbone repeat units. In addition, there may be (1-2)-β- and (1-3)-α-linked mannosyl repeat units in the side chains. The doublet resonance at 4.53 ppm (C1 at 103.46 ppm) is assigned to H1 of a (1-3)-linked glycosyl

repeat unit internal to a linear (1-3)-β-glucan segment since H1 is coupled to H2 at 3.35 ppm (C2 at 73.70 ppm) with a coupling constant of 7.85 Hz. This is consistent with a β configuration, and H2 is coupled to H3 at 3.50 ppm (C3 at 76.35 ppm) (Lowman, D.W., et al., *New insights into the structure of (1-3,1-6)-β-D-glucan side chains in the Candida glabrata cell wall.* PLoS One, 2011. 6(11): p. e27614). The smaller doublet resonance at 4.57 ppm (C1 at 103.25 ppm) is assigned to H1 in a glycosyl repeat unit and is coupled to H2 at 3.55 ppm with a coupling constant of 7.85 Hz, consistent with a β configuration also, suggesting a (1-3)-β-linked glycosyl repeat unit at the end of the glucan segment attached to a different monomer. Specific resonance assignments for rhamnose and galactose were not made.

Supplementary Figure 7b. COSY (left) and HSQC (right) 2D NMR Spectra of HMW-2. The COSY (proton-proton homonuclear correlation spectroscopy) 2D NMR spectrum provides correlations between neighboring protons H1 (the anomeric proton) and H2 to enable characterization of mannosyl repeat units in some of the structural motifs in HMW-2. The HSQC (proton-carbon heteronuclear correlation spectroscopy) 2D NMR spectrum furthers those correlations from protons H1 and H2 to their attached carbons (C1 and C2, respectively). Due to the similarity of the 1D NMR spectra of HMW-1 and HMW-2, COSY and HSQC 2D NMR spectra of HMW-1 were not collected. Structural motif assignments are solely based on the 2D NMR spectra for HMW-2.

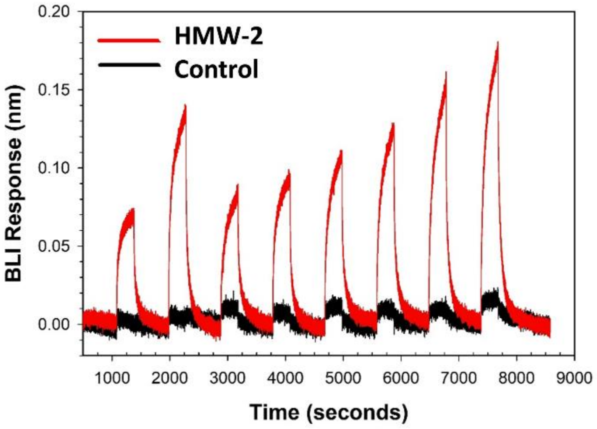

Supplementary Figure 8. Representative biolayer interferometry (BLI) sensorgram showing the interaction of HMW-2 with rhDectin-2. The HMW-2-rhDectin-2 interaction is shown in red. The control (shown in black) is a biosensor without rhDectin-2. We observed a dose response relationship over a HMW-2 concentration range of 3.125 to 400 µg/ml. Each experiment was repeated 3 – 6 times.

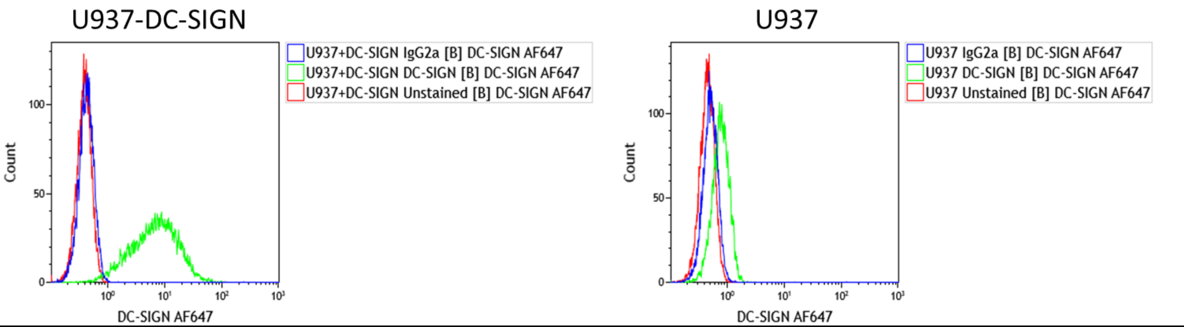

Supplementary Figure 9A. Flow cytometric analysis of DC-SIGN expression on U937-DC-SIGN cells.
U937-DC-SIGN and U937 cells were stained with anti-DC-SIGN-AF647 (green) or its corresponding isotype control (IgG2a-AF647, blue). Unstained cells (red) were used as a control. A representative histogram is presented from N=3.

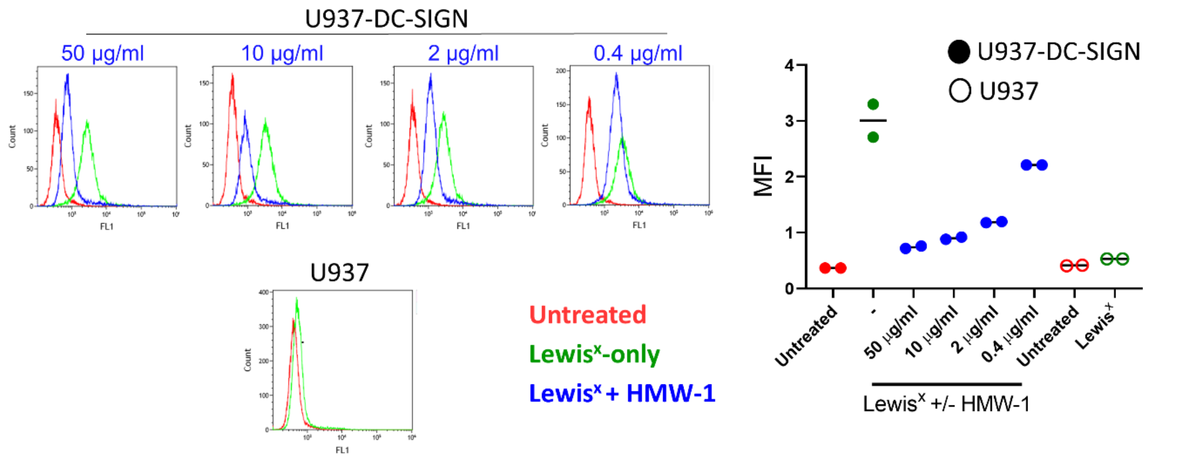

Supplementary Figure 9B. HMW-1 inhibits association of Lewis^X with U937-DC-SIGN cells in a dose dependent manner.
U937-DC-SIGN cells were pre-incubated with different concentrations of HMW-1 [50 µg/ml, 10 µg/ml, 2 µg/ml or 4 µg/ml] and then treated with 5 µg/ml of Lewis^x-PAA-FITC for 1 h. After incubation cell suspensions were analysed by flow cytometry. U937-DC-SIGN specifically associated with Lewis^x polymers and this association could be inhibited by the presence of HMW-1. U937 cells did not associate with Lewis^x polymers. Graph represents MFI of samples from a single experiment done in duplicate.

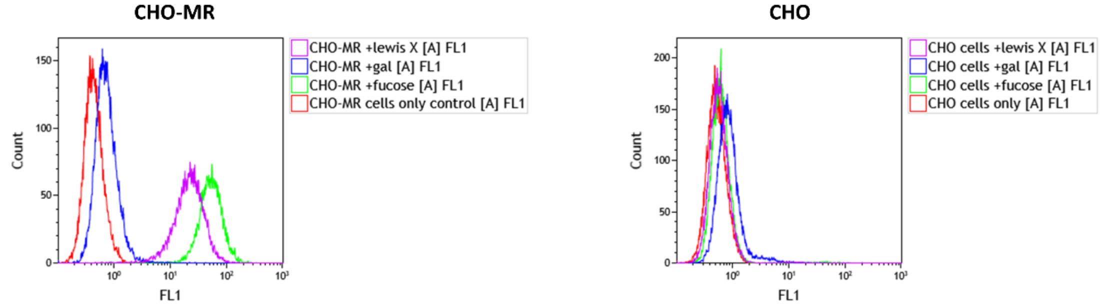

Supplementary Figure 9C. Association of fluorescently labelled polymeric ligands to

CHO-MR cells.
CHO-MR and CHO cells were treated with 5 µg/ml of Lewis^x-PAA-FITC (magenta), Fucose-PAA-FITC (green) or Galactose-PAA-FITC (blue) for 1 h. Cells without the addition of polymeric ligands (red) were used as controls. After incubation cells were trypsinised, fixed, and analysed by flow cytometry. Representative histograms from N=3 independent experiments (each done in duplicate).

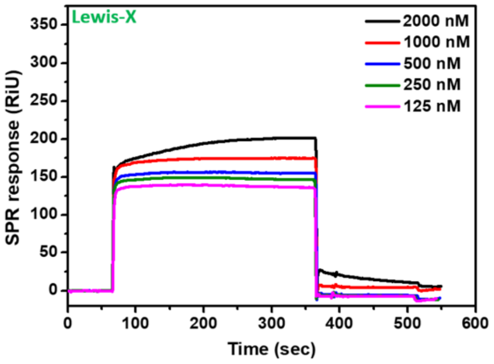

Kinetic data

	k_a (1/Ms)	k_d (1/s)	K_A (1/M)	K_D (M)	R_{max}
Lewis^X-PAA	4.31e^4	9.92e^{-3}	4.35e^6	2.30e^{-7}	200

Supplementary Figure 10. Analysis of human MR binding to Lewis^x-PAA using surface plasmon resonance.

Purified human MR (0.025 mg/ml, R&D systems) was immobilized via a standard amino coupling protocol onto a CM5 sensor chip that was activated by flowing a 1:1 mixture of 0.1 M N-hydroxysuccinimide and 0.1 M N-ethyl-N'-(dimethylaminopropyl)carbodiimide over the chip for 6 min at 25 °C at a flow rate of 20 µL/min after system equilibration with HEPES filtered buffer (10 mM HEPES pH 7.4, 150 mM NaCl, 0.005% Tween-20). Subsequently, channels 1 (blank) and 2 were blocked by following a solution of ethanolamine (1 M pH 8.5) for 10 min at 5 µL/min to remove remaining reactive groups on the channels. Sample solutions were prepared at varying concentrations (2000 nM-125 nM) in the same HEPES buffer to calculate the binding kinetics. Sensorgrams for each glycopolymer concentration were recorded with a 300 seconds injection of polymer solution (on period) followed by 150 seconds of buffer alone (off period). Regeneration of the sensor chip surfaces was performed using 10 mM HEPES buffer. Kinetic data was evaluated using a single set of sites (1:1 Langmuir Binding) model.

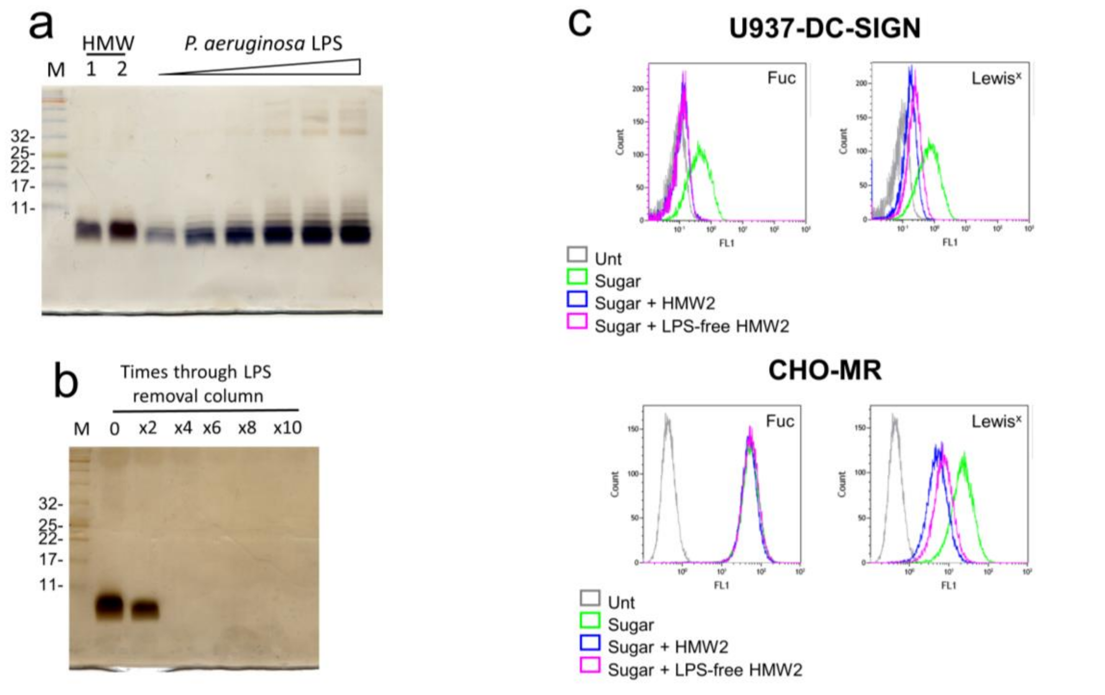

Supplementary Figure 11. Characterisation of HMW carbohydrate preparations.
a. Estimation of LPS contamination in HMW-1 and HMW-2 by SDS-PAGE and silver staining. HMW-1 and HMW-2 (37.5 µg) were electrophoresed under reducing conditions alongside different amounts of purified *P. aeruginosa* LPS (1, 2, 4, 6, 8 and 10 µg, Sigma L8643) in a 15% SDS-PAGE and silver stained using PlusOne Silver Staining kit, (71-71777-00, GE Healthcare). HMW-1 contained approx. 2 µg/37.5 µg. HMW-2 contained approx. 4 µg/37.5 µg. Molecular weight markers: Color Prestained Protein Standard, Broad range (11-245 kDa). **b.** Successful removal of LPS contamination in HMW-2 through repeated passages through a LPS-removal column (Pierce ™ High Capacity Endotoxin Removal Spin Columns (0.5 ml) Cat No 88274). To ensure lack of contamination 10 passages were performed. Successful LPS elimination was confirmed using huTLR4-reporter cells (data not shown). **c.** LPS removal does not affect the ability of HMW-2 to inhibit ligand internalisation by DC-SIGN and MR expressing cells. U937-DC-SIGN cells (top) or CHO-MR cells (bottom) were treated with HMW-2 (10 µg/ml, untreated or LPS-free) for 1 h, then fluorescently labelled sugar polymers (5 µg/ml) were added for a further 1 h. Association of fluorescent sugars to cells was measured by flow cytometry. Histograms show cell fluorescence intensity under different conditions. N=1. Fuc and Lewis^x refer to polymers bearing fucose or Lewis^x, respectively.

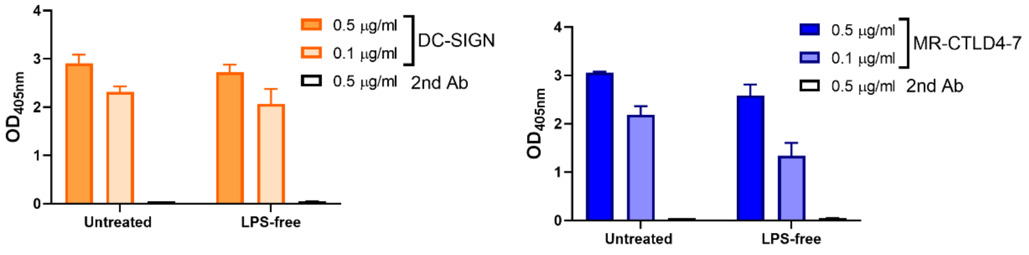

Supplementary Figure 12. Binding of DC-SIGN and MR-CTLD4-7 to HMW-2 is maintained after LPS removal.
Maxisorp plates were coated with Untreated or LPS-free HMW-2 (0.5 and 0.1 µg/ml) and incubated with DC-SIGN-Fc or MR-CTLD4-7-Fc. Binding was detected with anti-human Fc antibody conjugated to alkaline phosphatase. N=2 in duplicate.

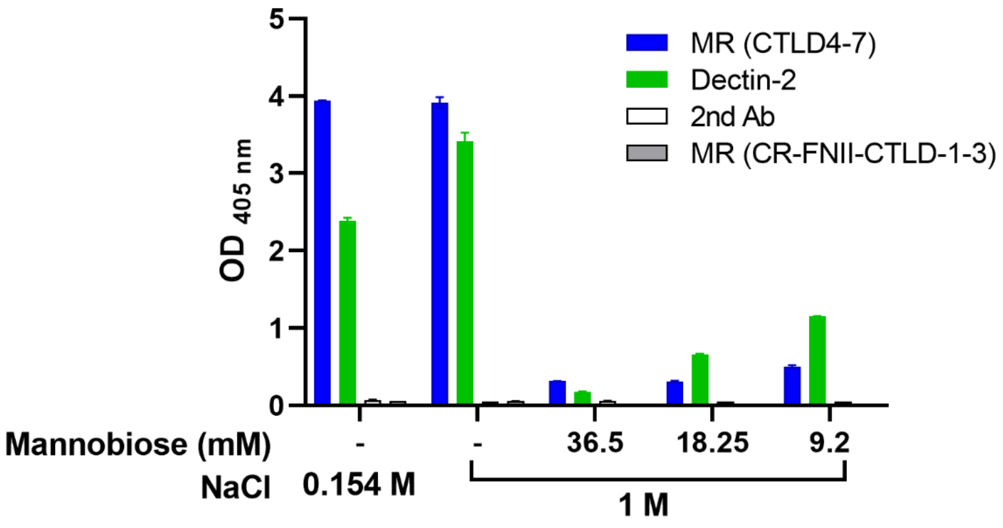

Supplementary Figure 13. Binding of MR-CTLD4-7 and Dectin-2 to LPS-free HMW-2 could be inhibited by α1-2 mannobiose.

Maxisorp plates were coated with LPS-free HMW-2 (0.5 µg/ml) and incubated with MR-CTLD-4-7-Fc or Dectin-2-Fc followed by anti-human Fc conjugated to alkaline phosphatase. Binding assays were performed at two NaCl concentrations (0.154 M and 1 M) and for the 1 M condition, incubation with Fc chimeric proteins was performed in the presence or absence of different concentrations of mannobiose (36.5, 18.25 and 9.2 mM). 1 M NaCl appeared to promote Dectin-2-Fc binding. Presence of competitor reduced binding of both Dectin-2-Fc and MR-CTLD4-7-Fc to HMW-2. N=2 in duplicate. Graph shows mean +/-SEM.

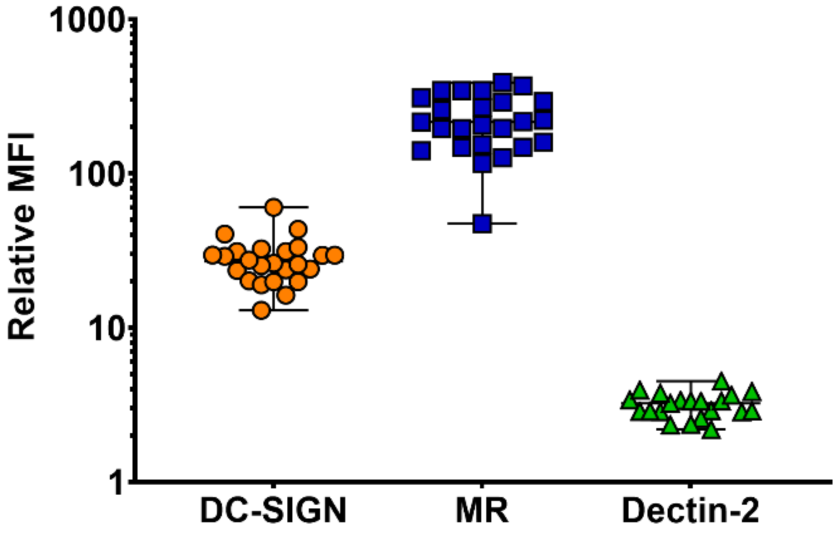

Supplementary Figure 14. Analysis of DC-SIGN, MR and Dectin-2 expression by moDCs.

Human moDCs generated from CD14+ monocytes in the presence of GM-CSF and IL-4 for 6 days were tested for MR and DC-SIGN expression by flow cytometry. MoDCs were re-suspended in X-vivo-15 media in a 15 ml falcon tube at a density of 1×10^6 cells/ml, centrifuged at 350 x g, for 5 min, acceleration and deceleration 3, at 4°C and re-suspended in 1.5 ml of 5% (v/v) mouse serum (M5905, Sigma) in PBA solution [0.5% (w/v) BSA and 0.1% (w/v) sodium azide (S2002, Sigma) prepared in PBS], and incubated for 1 h at 4°C. After washing with cold PBA three times, the cell density was adjusted to 50,000 cells/45µl of PBA and cells were incubated with mouse anti-human monoclonal antibodies or their matched isotype controls: CD206 (MMR)-Alexa Fluor 647 (AF647) (321116, Biolegend), DC209 (DC-SIGN)-AF647 (330112, Biolegend), or Dectin-2-Allophycocyanin (APC) (FAB3114A, R&D Systems) for 1 h at 4°C (1:10 dilution). Cells were washed twice with cold PBA, re-suspended in fresh cold PBA and fixed with 0.5% (v/v) paraformaldehyde in PBS, 4°C. Samples were analysed using a FC500 Beckman Coulter flow cytometer; 10,000-20,000 events were collected. Data were analysed using Kaluza Software 1.5a. Data are from 25 independent preparations (donors) and show consistent expression of both MR and DC-SIGN in all instances.

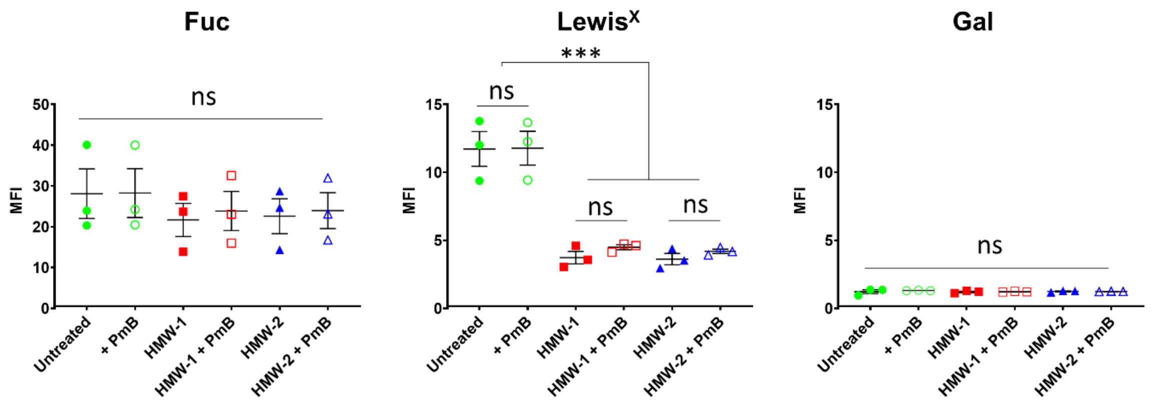

Supplementary Figure 15. Polymyxin B does not affect HMW-1- and HMW-2-mediated inhibition of Lewis^x uptake by moDCs.
Human moDCs were treated with HMW-1 or HMW-2 (10 µg/ml) for 1 h in the presence or absence of Polymyxin B (100 µg/ml, PmB), then fluorescently labelled sugar polymers (5 µg/ml) were added for a further 1 h. Association of fluorescent sugars to cells was measured by flow cytometry. Figure depicts mean ± SEM of median fluorescence intensity (MFI), N=3. Fuc, Lewis^x and Gal refer to polymers bearing fucose, Lewis^x or galactose, respectively. Data analysed using One-way ANOVA correcting for multiple comparisons using the Tukey's multiple comparison test. *** $p < 0.001$.

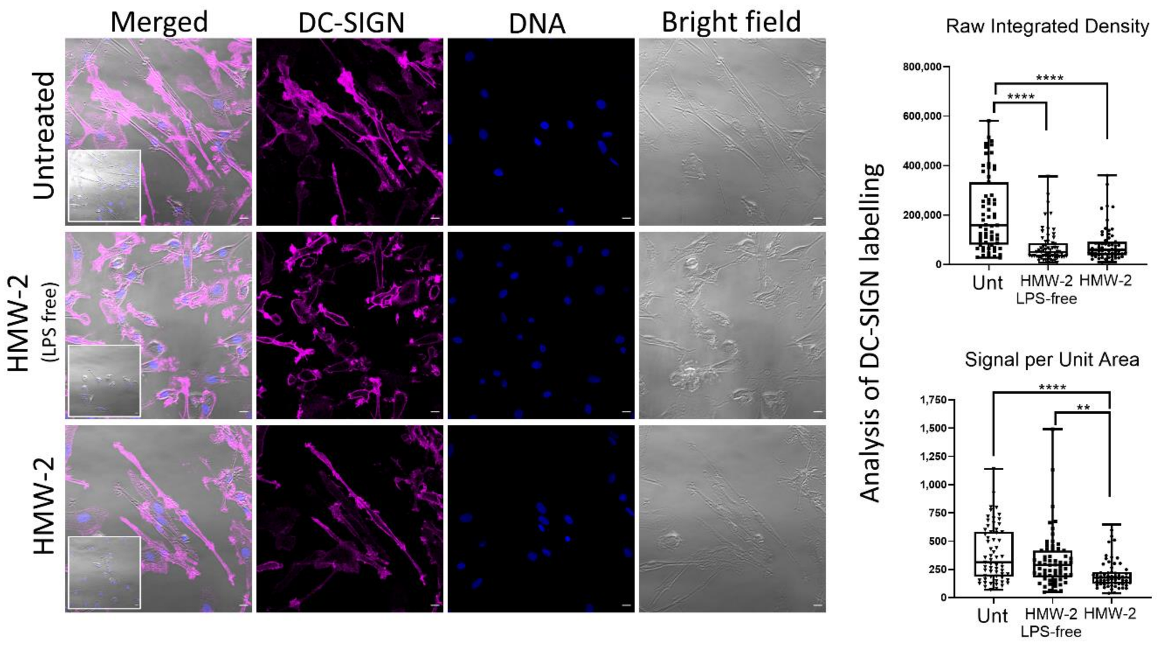

Supplementary Figure 16. Specific detection of DC-SIGN in human DCs in the presence and absence of HMW-2 and analysis of DC-SIGN surface expression in the presence of biofilm carbohydrates.

Left panel: HMW-2 (with and without LPS contamination) diluted in X-Vivo-15 medium (10 µg/ml) was used to coat chambers of µ-slide VI 0.4 flow slides for 24 h. moDCs (5 x 10^5 cells per channel) were added and incubated for 24 h. Samples were then fixed, permeabilised, and stained for DC-SIGN (magenta) and nucleus (blue). Insets show labelling with secondary antibody only. Scale bars: 10 µm.

Right panel: Cells were analysed for changes in DC-SIGN labelling intensity (Raw Integrated Density and Signal per Unit Area) in non-permeabilised cells. Data derive from 3 independent experiments, 20 cells per experiment were analysed. Statistical significance assessed using Kruskal-Wallis test corrected for multiple comparison using a Dunn's multiple comparison test.

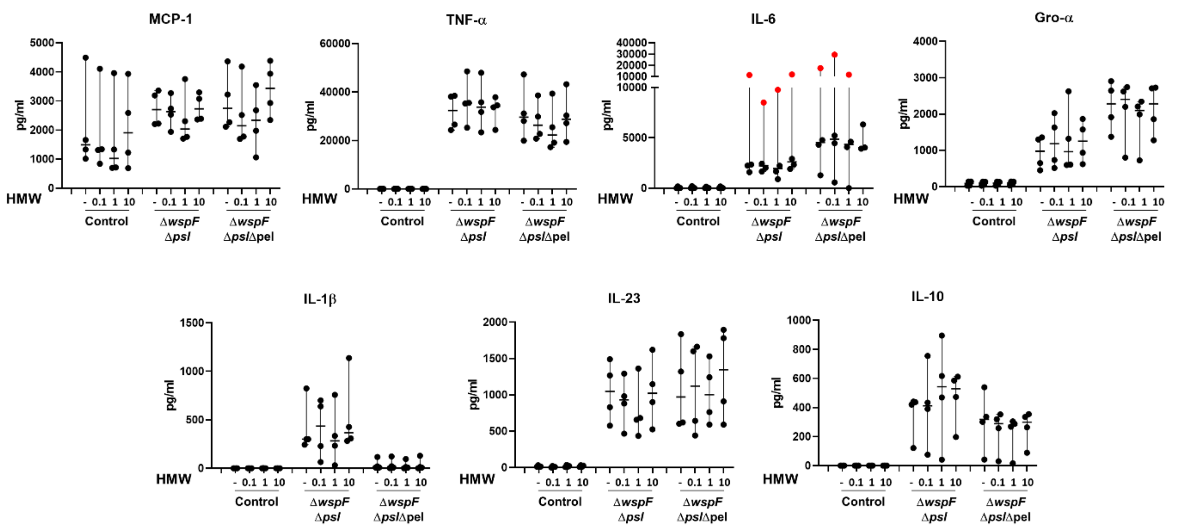

Supplementary Figure 17. LPS-free HMW-2 does not affect cytokine production by moDCs in response to Psl-deficient biofilms or planktonic cultures.

Cultures of Δ*wspF* Δ*psl* (biofilm-forming mutant lacking Psl) and Δ*wspF* Δ*psl* Δ*pel* (biofilm-deficient mutant lacking Psl and Pel) grown in 96-well tissue culture plates in X-Vivo15 medium for 24 h were fixed, pre-treated with different concentrations of LPS-free HMW-2 for 1 h (0.1, 1 and 10 µg/ml), and incubated with moDCs (5 x 10^4 cells per well) for 4 h in X-Vivo-15. Supernatants were then collected and analysed for cytokines. Graphs show median and range. N=4. Control represents moDCs only treated with LPS-free HMW-2. Red symbols relate to extrapolated high cytokine values. Cytokines were quantified using DuoSet ELISA (R&D Systems) and Magnetic luminex assay kits (R&D Systems).
