# Supplementary material for: Carbohydrates from Pseudomonas aeruginosa biofilms interact with immune C-type lectins and interfere with their receptor function
Source: NPJ Biofilms Microbiomes. 2021 Dec 8;7:87. doi: 10.1038/s41522-021-00257-w (PMC8655052; doi:10.1038/s41522-021-00257-w)
Supplement: Supplementary file 2 — Reporting Summary [file 41522_2021_257_MOESM2_ESM.pdf]

nature portfolio

Corresponding author(s): Luisa Martinez-Pomares
Miguel Camara

Last updated by author(s): Oct 18, 2021

Reporting Summary

Nature Portfolio wishes to improve the reproducibility of the work that we publish. This form provides structure for consistency and transparency in reporting. For further information on Nature Portfolio policies, see our Editorial Policies and the Editorial Policy Checklist.

Statistics

For all statistical analyses, confirm that the following items are present in the figure legend, table legend, main text, or Methods section.

n/a	Confirmed	
☐	☒	The exact sample size (n) for each experimental group/condition, given as a discrete number and unit of measurement
☐	☒	A statement on whether measurements were taken from distinct samples or whether the same sample was measured repeatedly
☐	☒	The statistical test(s) used AND whether they are one- or two-sided<br>*Only common tests should be described solely by name; describe more complex techniques in the Methods section.*
☒	☐	A description of all covariates tested
☐	☒	A description of any assumptions or corrections, such as tests of normality and adjustment for multiple comparisons
☐	☒	A full description of the statistical parameters including central tendency (e.g. means) or other basic estimates (e.g. regression coefficient) AND variation (e.g. standard deviation) or associated estimates of uncertainty (e.g. confidence intervals)
☐	☐	For null hypothesis testing, the test statistic (e.g. F, t, r) with confidence intervals, effect sizes, degrees of freedom and P value noted<br>*Give P values as exact values whenever suitable.*
☒	☐	For Bayesian analysis, information on the choice of priors and Markov chain Monte Carlo settings
☒	☐	For hierarchical and complex designs, identification of the appropriate level for tests and full reporting of outcomes
☒	☐	Estimates of effect sizes (e.g. Cohen's d, Pearson's r), indicating how they were calculated

Our web collection on statistics for biologists contains articles on many of the points above.

Software and code

Policy information about availability of computer code

Data collection	*Provide a description of all commercial, open source and custom code used to collect the data in this study, specifying the version used OR state that no software was used.*
Data analysis	Data analysis was done using PGraphPad Prism, Fiji and Kaluza

For manuscripts utilizing custom algorithms or software that are central to the research but not yet described in published literature, software must be made available to editors and reviewers. We strongly encourage code deposition in a community repository (e.g. GitHub). See the Nature Portfolio guidelines for submitting code & software for further information.

Data

Policy information about availability of data

All manuscripts must include a data availability statement. This statement should provide the following information, where applicable:

- Accession codes, unique identifiers, or web links for publicly available datasets
- A description of any restrictions on data availability
- For clinical datasets or third party data, please ensure that the statement adheres to our policy

Authors can confirm that all relevant data are included in the paper and its supplementary information files.

Field-specific reporting

Please select the one below that is the best fit for your research. If you are not sure, read the appropriate sections before making your selection.

☒ Life sciences ☐ Behavioural & social sciences ☐ Ecological, evolutionary & environmental sciences

For a reference copy of the document with all sections, see nature.com/documents/nr-reporting-summary-flat.pdf

Life sciences study design

All studies must disclose on these points even when the disclosure is negative.

Sample size	Not relevant
Data exclusions	No data were excluded from analyses.
Replication	All experiments were replicated at least twice, in most case more often. Duplicates or triplicates (technical repeats) were run within each experimental repeat.
Randomization	*Describe how samples/organisms/participants were allocated into experimental groups. If allocation was not random, describe how covariates were controlled OR if this is not relevant to your study, explain why.*
Blinding	Not relevant

Reporting for specific materials, systems and methods

We require information from authors about some types of materials, experimental systems and methods used in many studies. Here, indicate whether each material, system or method listed is relevant to your study. If you are not sure if a list item applies to your research, read the appropriate section before selecting a response.

Materials & experimental systems

n/a	Involved in the study	
☐	☒	Antibodies
☐	☒	Eukaryotic cell lines
☒	☐	Palaeontology and archaeology
☒	☐	Animals and other organisms
☐	☒	Human research participants
☒	☐	Clinical data
☒	☐	Dual use research of concern

Methods

n/a	Involved in the study	
☒	☐	ChIP-seq
☐	☒	Flow cytometry
☒	☐	MRI-based neuroimaging

Antibodies

Antibodies used	Goat anti-rabbit IgG conjugated to alkaline phosphatase diluted 1:2000 (A3687, Sigma)<br>Recombinant proteins MR-CTLD4-7 (CTLD-4-7-Fc, prepared in house, (ref:35)), DC-SIGN (DC-SIGN-Fc, R&D Systems) and Dectin-2 (Dectin-2-Fc, Enzo)<br>Goat anti-Human IgG conjugated to Alexa fluor 647 (A21445, Invitrogen)<br>Recombinant human MR (CD206) and Dectin-2 with a poly-His-tag were from R&D Systems (Minneapolis, MN). Biotinylated DC-SIGN was generated as described (Reference 18).<br>Rabbit polyclonal antibody against DC-SIGN (ab5715, Abcam)<br>Alexa Fluor 647-AffiniPure Donkey anti-Rabbit IgG (H+L) (711-605-152, Jackson ImmunoResearch).<br>For Flow cytometry analysis of moDCs: anti-human monoclonal antibodies or their matched isotype controls: CD206 (MMR)-Allophycocyanin (APC) (321109, Biolegend), DC209 (DC-SIGN)-APC (330107, Biolegend), CLEC6A (Dectin-2)-APC (FAB3114A, R&D), CLEC7A (Dectin-1)-APC (144306, Biolegend), CD66b-FITC (REA306, Miltenyi Biotec), APC Mouse IgG1 (MR) Isotype Ctrl (400120, Biolegend), APC Mouse IgG2a (DC-SIGN) Isotype Ctrl (400222, Biolegend), APC Mouse IgG1 (Dectin-2) Isotype Ctrl (IC002A, R&D), APC Rat IgG1 (Dectin-1) Isotype Ctrl (400411, Biolegend) or human IgG1 FITC-REA (CD66b) control Ab (130-113-437, Miltenyi Biotec)
Validation	All assays were done with relevant negative controls.

Eukaryotic cell lines

Policy information about cell lines

Cell line source(s)	U937 cells transfected with human DC-SIGN (U937-DC-SIGN) and control U937 cells were obtained from the American Type Culture Collection (ATCC).<br>CHO and CHO-MR were described in reference 41.<br>Dectin-2 NFkB SEAP reporter cells (HEK-blue 293 muDectin-2) and control cells were from Invivogen.
Authentication	We established DC-SIGN expression in U937-DC-SIGN cells by FACS.<br>CHO and CHO-MR were described in reference 41.<br>Dectin-2 NFkB SEAP reporter cells (HEK-blue 293 muDectin-2) responded to Zymosan.
Mycoplasma contamination	Cells were not routinely tested for mycoplasm contamination.
Commonly misidentified lines<br>(See ICLAC register)	*Name any commonly misidentified cell lines used in the study and provide a rationale for their use.*

Human research participants

Policy information about studies involving human research participants

Population characteristics	Blood donors
Recruitment	Samples provided by Blood and Transplant NHS. Non clinical tissue. Buffy Coats.
Ethics oversight	Faculty of Medicine & Health Sciences Research Ethics Committee

Note that full information on the approval of the study protocol must also be provided in the manuscript.

Flow Cytometry

Plots

Confirm that:

☒ The axis labels state the marker and fluorochrome used (e.g. CD4-FITC).

☒ The axis scales are clearly visible. Include numbers along axes only for bottom left plot of group (a 'group' is an analysis of identical markers).

☐ All plots are contour plots with outliers or pseudocolor plots.

☐ A numerical value for number of cells or percentage (with statistics) is provided.

Methodology

Sample preparation	All cells tested by flow cytometry comprised a single cell population and were gated on viable cells. They were harvested by centrifugation.
Instrument	Beckman Coulter FC500 flow cytometer
Software	Kaluza
Cell population abundance	*Describe the abundance of the relevant cell populations within post-sort fractions, providing details on the purity of the samples and how it was determined.*
Gating strategy	Cells were gated on viable cells.

☐ Tick this box to confirm that a figure exemplifying the gating strategy is provided in the Supplementary Information.
